# Diverse Protein Profiles in CNS Myeloid Cells and CNS Tissue From Lipopolysaccharide- and Vehicle-Injected APP_SWE_/PS1_ΔE9_ Transgenic Mice Implicate Cathepsin Z in Alzheimer’s Disease

**DOI:** 10.3389/fncel.2018.00397

**Published:** 2018-11-06

**Authors:** Camilla Thygesen, Laura Ilkjær, Stefan J. Kempf, Anne Louise Hemdrup, Christian Ulrich von Linstow, Alicia A. Babcock, Sultan Darvesh, Martin R. Larsen, Bente Finsen

**Affiliations:** ^1^Department of Neurobiology, Institute of Molecular Medicine, University of Southern Denmark, Odense, Denmark; ^2^Department of Biochemistry and Molecular Biology, University of Southern Denmark, Odense, Denmark; ^3^Brain Research – Inter-Disciplinary Guided Excellence, Department of Clinical Research, University of Southern Denmark, Odense, Denmark; ^4^Department of Medicine (Neurology and Geriatric Medicine) – Department of Medical Neuroscience, Dalhousie University, Halifax, NS, Canada; ^5^Department of Chemistry and Physics, Mount Saint Vincent University, Halifax, NS, Canada

**Keywords:** Alzheimer’s disease, systemic inflammation, quantitative proteomics, microglia, perivascular cells, amyloid precursor protein, apolipoprotein E, cathepsin Z

## Abstract

Neuroinflammation, characterized by chronic activation of the myeloid-derived microglia, is a hallmark of Alzheimer’s disease (AD). Systemic inflammation, typically resulting from infection, has been linked to the progression of AD due to exacerbation of the chronic microglial reaction. However, the mechanism and the consequences of this exacerbation are largely unknown. Here, we mimicked systemic inflammation in AD with weekly intraperitoneal (i.p.) injections of APP_SWE_/PS1_ΔE9_ transgenic mice with *E. coli* lipopolysaccharide (LPS) from 9 to 12 months of age, corresponding to the period with the steepest increase in amyloid pathology. We found that the repeated LPS injections ameliorated amyloid pathology in the neocortex while increasing the neuroinflammatory reaction. To elucidate mechanisms, we analyzed the proteome of the hippocampus from the same mice as well as in unique samples of CNS myeloid cells. The repeated LPS injections stimulated protein pathways of the complement system, retinoid receptor activation and oxidative stress. CNS myeloid cells from transgenic mice showed enrichment in pathways of amyloid-beta clearance and elevated levels of the lysosomal protease cathepsin Z, as well as amyloid precursor protein, apolipoprotein E and clusterin. These proteins were found elevated in the proteome of both LPS and vehicle injected transgenics, and co-localized to CD11b^+^ microglia in transgenic mice and in primary murine microglia. Additionally, cathepsin Z, amyloid precursor protein, and apolipoprotein E appeared associated with amyloid plaques in neocortex of AD cases. Interestingly, cathepsin Z was expressed in microglial-like cells and co-localized to CD68^+^ microglial lysosomes in AD cases, and it was expressed in perivascular cells in AD and control cases. Taken together, our results implicate systemic LPS administration in ameliorating amyloid pathology in early-to-mid stage disease in the APP_SWE_/PS1_ΔE9_ mouse and attract attention to the potential disease involvement of cathepsin Z expressed in CNS myeloid cells in AD.

## Introduction

Neuroinflammation is a hallmark of Alzheimer’s disease (AD) pathology, and is considered a major contributor to AD pathogenesis (Zhang et al., 2013; Heneka et al., 2015). Onset of inflammation is closely linked to, and may even precede, the development of other neuropathological characteristics of AD (Heppner et al., 2015). These include the deposition of amyloid-beta (Aβ) into plaques in the neuropil, the aggregation of hyper-phosphorylated tau into tangles, as well as synaptic and neuronal degeneration (Heneka et al., 2015). Microglia, the brain innate immune cells, are considered the main drivers of inflammatory reactions in the brain (Heppner et al., 2015). Activated microglia produce inflammatory molecules, such as tumor necrosis factor (TNF), and interleukin 1β (IL1β) (Eggen et al., 2013), molecules which have been shown to be increased in AD brains along with multiple other inflammatory molecules (Griffin et al., 1989; Bauer et al., 1991; Tarkowski et al., 2003). Markers of inflammation, such as C-reactive protein and TREM2, are also elevated in blood and/or cerebrospinal fluid years before the onset of dementia (Schmidt et al., 2002; Suárez-Calvet et al., 2016; Lai et al., 2017). In addition, epidemiological studies have shown an effect of non-steroidal anti-inflammatory drugs in the risk of developing AD, however, beneficial effects have not been replicated in randomized clinical trials (Deardorff and Grossberg, 2017).

There is evidence that systemic inflammation, typically caused by infection, may additionally contribute to the progression of AD (Cunningham, 2013). Thus, the number of infections and infection-induced delirium have both been associated with increased incidence of dementia (Rahkonen et al., 2000; Holmes et al., 2009). A major cause of infection is Gram-negative bacteria and the blood levels of lipopolysaccharide (LPS), which is a Gram-negative bacterial cell wall protein and an endotoxin, has been found increased in AD cases (Zhan et al., 2018). In addition, LPS has been detected in both gray and white matter of AD brains, accumulating in amyloid plaques (Zhan et al., 2018). Studies using peripheral LPS administration to stimulate neuroinflammation in mouse models of AD have been contradictory with regard to the effect of LPS on the amyloid pathology (Cunningham, 2013). Administration of LPS by the intraperitoneal (i.p.) route activates the innate immune system both in the periphery and centrally. Centrally, LPS exacerbates age-associated changes in microglial activation state and it causes oxidative stress and neuronal death in mouse models of neurodegeneration and AD (Sly et al., 2001; Cunningham, 2005; Godbout, 2005; Lee et al., 2008). Peripheral administration of LPS has mainly been shown to accentuate Aβ pathology, however, some studies have shown beneficial effects of LPS after both peripheral and central administration, resulting in lower Aβ-levels in mouse models of AD, an effect considered to be microglial-dependent (DiCarlo et al., 2001; Quinn et al., 2003; Herber et al., 2007).

The purpose of this study was (1) to investigate the effect of repeated systemic administration of LPS on amyloid pathology in the APP_SWE_/PS1_ΔE9_ transgenic (Tg) mouse model of AD, and anticipating that effects of LPS on Aβ pathology would be microglial-dependent, to (2) define the microglial disease associated proteome by proteomic profiling of CD11b^+^ central nervous system (CNS) myeloid cells from 24-month-old Tg and wild type (Wt) mice and tissue from the LPS- and vehicle-injected Tg and Wt mice. Wild type and Tg mice received weekly i.p. injections with LPS or vehicle from the age of 9 to 12 months, corresponding to the period when the increase in neocortical Aβ plaque load shows its steepest increase (Babcock et al., 2015). One hemisphere was used for stereological estimation of Aβ plaque load, while the contralateral neocortex and hippocampus were used for quantitative studies of Aβ levels and pro-inflammatory cytokine mRNA, and for quantitative proteomics studies, respectively. The CD11b^+^ cell proteome was used to select CNS myeloid cell, particularly microglial, proteins influenced by LPS administration in the Tg mice. We report that repeated LPS-administration mitigates neocortical Aβ pathology while exacerbating the neuroinflammatory reaction. Protein pathways influenced by the LPS-administration in the Tg mice included retinoic receptor activation, redox reactions and the complement system. CNS myeloid cell proteins overlapping with proteins up-regulated in Tg mice included the AD markers amyloid precursor protein (APP), apolipoprotein E (APOE), and Clusterin (Clu) as well as the lysosomal protease cathepsin Z (Ctsz).

## Materials and Methods

### Animals

Experiments were performed according to protocol number J.nr. 2011/561-1950 and project Id. 2015-15-0202-00037 approved by the National Danish Animal Research Committee. APP_SWE_/PS1_ΔE9_ Tg and Wt littermate mice bred on a C3H/HeN × C57BL/J background, were housed in the Biomedical Laboratory at the University of Southern Denmark under a 12:12 h light:dark cycle (lights on 6.30 am) with food and water available *ad libitum*. C57BL/6J mice from Taconic (DK) were used for the breeding of mouse pups.

### Human Neocortex Tissue

Autopsy brain samples of frontal cortex were obtained from AD and control cases from The Maritime Brain Tissue Bank, Dalhousie University, Halifax, NS, Canada following approval from the Danish Biomedical Research Ethical Committee for the Region of Southern Denmark and the Capital Health Research Ethics Board in Canada. Informed consent was obtained for all donors. Brains were removed ≤24 h post-mortem, immersion fixed in 10% formalin in phosphate buffer (pH 7.4) and transferred to 40% sucrose with 0.6% sodium azide. Five definite AD cases (three females and two males) and three control cases (two females and one male) were included in the study. The two groups were age-matched (AD: 85 ± 1.1 years, Con: 79 ± 9.1 years) (Supplementary Table S1). AD was confirmed based on Khachaturian criteria (Khachaturian, 1985), following neuropathological examination of paraffin sections from neocortex, basal forebrain, amygdala, hippocampus, entorhinal cortex, basal ganglia, thalamus and mid-brain using HE and Bielschowsky silver stain, and immunohistochemistry for Aβ, α-synuclein, tau and ubiquitin. Tissue blocks were processed into 50 μm thick free-floating sections in a vibratome (Leica, Denmark), which were stored in a cryoprotective solution at −14°C until used for staining.

### LPS Administration

A total of 37 9-month-old female Tg and Wt were used in this experiment (*n* = 6–15/group). LPS (*E. coli* 0111:B4 Sigma) was used in a concentration of 0.05 mg/mL sterile phosphate buffered saline (PBS). Mice were administered LPS (0.5 mg/kg equivalent to 250 endotoxin units/g) or an equal volume of PBS i.p. once a week for 13 weeks. PBS-administered mice were weighed before injection. LPS-administered mice were weighed and monitored for sickness behavior before injection, 4-, and 24-h after injections. LPS- and PBS- administered mice did not differ in body weight. Two LPS-administered mice died during the experiment. All mice were sacrificed 4 h after the final administration. To establish a baseline for the Aβ pathology, four 9-month-old female Tg mice were included in the experiment.

### Tissue Processing

Mice were given an overdose of pentobarbital prior to perfusion with 20 mL ice-cold PBS, where after brains were isolated. The right neocortex was split in thirds and frozen on dry ice along with the hippocampus for RNA and protein isolation, while the left hemisphere was either frozen in CO_2_ snow (*n* = 1–2/group) or immersed in 4% paraformaldehyde (PFA) overnight (ON), 1% PFA ON, and finally 20% sucrose ON, and frozen in CO_2_ snow for histology. The hemibrains were sectioned in a cryostat into 30 μm thick coronal sections.

### Isolation of CNS Myeloid Cells

Twenty-four-month-old, male Tg and C57BL/6 mice (*n* = 6–8/group) were PBS-perfused, brains were removed, the meninges were stripped and the neocortex and hippocampus were isolated and digested with TrypSelect/DNAse and homogenized with a 70 μM cell strainer. The cells were isolated by a Percoll density gradient, and myelin was aspirated before addition of magnetic CD11b micro-beads. The cell suspension was loaded onto a MACS^®^ column, placed in a MACS separator and CD11b^+^ cells were isolated per the manufacturer’s instruction.

### Primary Murine Microglia

Neonatal primary microglia were harvested from mixed glial cultures using the method by Krabbe et al. (2012). Postnatal day 0–3 C57BL/6J pups were decapitated and brains were isolated in ice-cold Hank’s balanced salt solution (HBSS) (1x, Life Technologies) and treated with trypsin (Worthington) and DNAse (Biochrom). Enzyme activity was quenched by adding complete medium [Dulbecco’s modified eagle medium (DMEM), 10% fetal bovine serum (FBS), and 1% penicillin/glutamine/streptomycin (PGS), Life Technologies]. Next, brains were homogenized and centrifuged for 10 min at 800RPM in 4°C. Pellet was re-suspended in complete medium and transferred to filter-free T75 flasks (Orange Scientific) coated with poly-L-lysine (Sigma) and incubated in a 37°C humid atmosphere. After 2 days, cells were rinsed in Dulbecco’s phosphate-buffered saline (D-PBS) (1x Life Technologies) and provided fresh complete medium. After five additional days, medium was replaced with complete medium and conditioned L929 fibroblast medium. Cells were incubated for additional 2 days and microglia were harvested by shake-off at 100–130 RPM for 30 min. Microglia were harvested three times from the mixed glial cultures with 2 days interval, counted using a Bürker-Türk counting chamber and plated in 24-well culture plates at a density of 1.5 × 10^6^ cells/mL. The culture purity was >99%, assessed by flow cytometry.

### Mass-Spectrometry (MS) Based Proteomics

The complete sample processing workflow is depicted in Supplementary Figures S1A,B. Table 1 shows the number of protein quantifications and significantly regulated proteins in each condition. The complete sample processing protocol can be seen in the Supplementary Materials and Methods. Hippocampal samples for proteomics were processed as described in Kempf et al. (2016) and Thygesen et al. (2018). Briefly, snap-frozen hippocampal tissue was Dounce homogenized in 8M urea and CD11b^+^ cell proteins were isolated by the Qiagen AllPrep kit for simultaneous isolation of protein, RNA and DNA. Samples were reduced, alkylated and digested with Lys-C and trypsin. A total of 100 μg peptides per hippocampal samples were desalted with R2/R3 columns and labeled using iTRAQ-8plex (AB Sciex). A total of 24 mice (*n* = 6/group) were used for the proteomics analysis of the hippocampus, resulting in six biological replicates, which were labeled using three iTRAQ-8plex kits according to the manufacturer’s instruction. CD11b^+^ samples were labeled using an iTRAQ 4-plex kit according to the manufacturer’s instruction. Samples were combined in equal ratios, desalted and fractionated using high-pH fractionation (hippocampus only) and hydrophilic interaction chromatography (HILIC) (hippocampus and CD11b^+^) as described in McNulty and Annan (2008) and Melo-braga et al. (2015). The fractionated peptides were analyzed by nano-LC online connected to an Orbitrap Fusion tribrid mass spectrometer (Thermo Fischer Scientific). For details on mass spectrometry settings see the Supplementary Materials and Methods. Raw data was analyzed using Proteome Discoverer (V1.4.1.14, Thermo Fischer Scientific). Precursor mass tolerance of 10 ppm, product ion mass tolerance of 0.02 Da. Fixed modifications included carbamidomethylation of Cys and iTRAQ8-plex labeling for Lys and N-termini. Quantification was performed on the centroid peak intensity with the “reporter ions quantifier” node. The Mascot Percolator algorithm was used with a *q*-value filter of 0.01 together with Mascot and Sequest HT peptide rank 1, Mascot score > 22 and Sequest HT ΔCn of 0.1. Moreover, a cut-off value of Xcorr score for charge states of +1, +2, +3, and +4 higher than 1.5, 2, 2.25, and 2.5, respectively, were considered for further analysis and filtered for a FDR of 0.01. Proteins were identified with at least two unique peptides. For hippocampus samples the statistical analysis was performed on log_2_ transformed quantification values by a moderated *t*-test (limma) using rank products, with a *q*-value threshold of 0.01, corrected for statistical error by means of multiple testing (Schwämmle et al., 2013). For CD11b^+^ samples significantly regulated proteins were determined based on expression ratios being outside two standard deviations of the biological replicates, cut-off values of 1.3 and 0.75 based on the average experimental technical variance of brain technical replicates in mass spectrometry (Kempf et al., 2014, 2016).

**Table 1:** Numbers of quantified and regulated proteins in the hippocampal proteome of LPS- and PBS-injected mice and in the CD11b^+^ cell proteome from Tg and Wt mice.

Region and cells	Quantified proteins	Regulated proteins	
Hippocampal proteome	2653	Tg LPS vs. Tg PBS	19
		Wt LPS vs. Wt PBS	0
		Tg LPS vs. Wt LPS	17
		Tg PBS vs. Wt PBS	11
CD11b^+^ cell proteome	467	Tg vs. Wt	52

The MS proteomics data have been deposited to the ProteomeXchange Consortium^1^ via the PRIDE partner repository (Vizcaíno et al., 2012) with the dataset identifier <pxd005785>.

#### Bioinformatics Analysis of Protein Classes and Affected Signaling Pathways

Significantly regulated proteins were analyzed by Ingenuity^®^ Pathway Analysis (IPA^®^) (to annotate proteins implicated in canonical pathways using Fisher’s exact test, *p* < 0.05) and gene ontology (GO) enrichment analysis was performed by the Database for Annotation, Visualization and Integrated Discovery (DAVID) software version 6.8, Functional annotation chart, using the default modes with a modified Fisher’s exact test (EASE score threshold of 0.1) counts threshold of 2, *p* < 0.05 (Huang et al., 2009). Proteins were analyzed based on GO enrichment of molecular pathways, cellular components, biological processes and KEGG pathways.

### Immunohistochemical (IHC) and Immunofluorescence (IF) Staining

#### Primary Antibodies and General Procedures

Biotinylated mouse anti-human Aβ (Covance, clone 6e10), rat anti-mouse CD11b (AbD Serotec, clone 5C6), rabbit anti-mouse APOE (Abcam, clone EPR19392) rabbit anti-mouse Clu (Abcam, clone EPR17539-95), rabbit anti-mouse APP (Abcam, clone Y188), rabbit anti-mouse Ctsz (Abcam, clone EPR14357), rabbit-anti-mouse beta-hexosaminidase (Hexb) (Cloud-clone), mouse anti-human pTau (Thermo Scientific, clone AT8), rabbit anti-human Iba1 (WAKO) and mouse anti-human CD68 (DAKO, clone PG-M1). As substitution control was used rabbit IgG (Dako), biotinylated mouse IgG1 (Caltag), and rat IgG2b (Nordic Biosite). For additional details on the primary antibodies as well as secondary reagents, see Supplementary Table S2. Stainings were performed in a systematic way, staining sections from different mouse groups and from AD and non-AD cases in parallel under identical conditions, and with inclusion of substitution controls in all stainings.

#### Procedure for IHC Staining for Aβ and CD11b

Sections were stained using the protocol in Babcock et al. (2015). In the case of Aβ, sections were hydrated in TBS followed by a 30 min antigen retrieval in 70% formic acid. Next, sections were rinsed 10 min in TBS and 3×15 min in TBS/1% Triton-100 (TBST) and blocked with TBS/10% FBS for 30 min. Sections were incubated ON with primary antibody at 4°C (biotinylated mouse anti-human Aβ, Covance, clone 6e10). Next day, sections were rinsed in TBST and endogenous peroxidase activity was blocked with TBS:MeOH:H_2_O_2_ (8:1:1) for 10 min and washed in TBST. Sections were then incubated with streptavidin-conjugated HRP (SA-HRP, Ge Healthcare, United Kingdom) for 1 h at room temperature (RT) and rinsed in TBS. Sections were developed using 0.05% 3,3′-diaminobenzidine (DAB) with 0.01% H_2_O_2_ for 5 min. Finally, sections were rinsed in TBS, dehydrated in graded ethanol, cleared in xylene, and cover-slipped with Depex. No staining was observed when primary antibodies were omitted or substituted with the biotinylated isotype control.

#### Procedure for IHC Staining for APP, APOE, Clu, Ctsz, and HexB

Sections were brought to RT and rinsed in TBST for 15 min. Sections were blocked for 30 min with TBS/10% FBS and incubated 60 min with primary antibodies at RT and then ON at 4°C. Next, sections were rinsed in TBST 3 × 15 min and incubated 60 min with a 1:5 mix of AP-conjugated anti-rabbit IgG antibody (Sigma, A3812) and mouse serum (DAKO, XO910) to reduce unspecific binding, followed by a 3 × 15 min rinse in TBS and development with a 5-bromo-4-chloro-3-indolyl phosphate (BCIP) and nitroblue tetrazolium (NBT) solution containing levamisol to block endogenous AP activity.

#### Procedure for IHC Staining of Human Tissue

Five AD and three control cases were stained for Aβ, pTau, Iba1, APOE, APP, Ctsz and Hexb (Supplementary Table S2). Vibratome sections were rinsed ON at 4°C in PBS. Next day sections were brought to RT and demasked by heating in Tris-EGTA buffer, pH 9.1. Sections were rinsed 30 min in TBS, endogenous peroxidase activity was blocked with TBS:MeOH:H_2_O_2_ (8:1:1) for 30 min and washed 3 × 30 min in TBST followed by a 60 min block in TBS/10% FBS. Sections were incubated with primary antibodies in TBS/10% FBS 60 min at RT and ON at 4°C. Then sections were rinsed 3 × 30 min in TBST and ON at 4°C in TBS. The following day sections were incubated 1.5 h with EnVision anti rabbit (DAKO, K4002), rinsed 3 × 30 min in TBS and developed using 0.05% DAB with 0.01% H_2_O_2_. Finally, sections were rinsed in TBS, dehydrated in graded ethanol, cleared in xylene, and cover-slipped with Depex.

#### Procedure for IF Staining

Cryostat sections, human vibratome sections or PFA-fixed primary microglial cells were stained using a combination of simultaneously applied primary antibodies to detect co-localization of Aβ plaques, APP, APOE, Clu, Ctsz, and Hexb with CD11b^+^ or CD68^+^ microglia. The procedure was as described above except that the primary antibodies were detected using a combination of AlexaFluor-488-labeled goat-anti-rabbit IgG (Invitrogen) and SA-TRITC (Invitrogen), AlexaFluor 594-labeled goat-anti rat (Invitrogen) or AlexaFluor 594-labeled goat-anti mouse, at which point the sections were kept in the dark. Nuclei were visualized by DAPI (Invitrogen, D3571), which was used in a concentration of 300 nM. Rabbit IgG, rat IgG2b and mouse IgG1 controls were included as described above. For human IF stainings, a step of autofluorescence quenching was performed after incubation with DAPI using an autofluorescence eliminator reagent (Merck Millipore, 2160), following manufacturers protocol.

#### Microscopy

Images of IHC stainings were captured with an Olympus BX51 microscope with an Olympus DP73 camera. IF images were captured with an Olympus BX63 upright, automated fluorescence microscope installed with an Olympus DP80 camera, X-cite 120LED system with filter cubes (U-FBNA FL Ex.BP470-495 Em.BA510-550, U-FGNA FL Ex.BP540-550 EM.BA575-625, U-FMCHE FL Ex.BP 565-585 Em.BA600-690, U-FUNA FL Ex.BP360-370 EmBA420-460), objectives: UPLSAPO2 10X/0.4, UPLSAPO2 40X/0.95, PLAPON0 60X/1.42 and UPLSAPO 100X/1.4 and CellSens Software. Images of tissues and cells were captured by creating a z-stack. For cells the z-stacks was made with the 60x/1.42 objective, refractive index (RI) of 1.518, xyz calibration 0.169 μm^∗^0.169 μm^∗^0.350 μm, the average z-stack height was 8 μm with a step-height of 0.32 μm. For mouse tissue the 60x/1.42 objective, RI of 1.518, xyz calibration 0.169 μm^∗^0.169 μm^∗^0.350 μm and the 100x/1.4 objective, RI of 1.518, xyz calibration 0.101 μm^∗^0.101 μm^∗^0.250 μm, the average z-stack height was 22 μm with a step-height of 0.29 μm. For human tissue the z-stacks were made with the 60x/1.42 objective, RI of 1.518, xyz calibration 0.169 μm^∗^0.169 μm^∗^0.250 μm and the 100x/1.4 objective, RI of 1.518, xyz calibration 0.101 μm^∗^0.101 μm^∗^0.250 μm, the average z-stack height was 20 μm with a step-height of 0.24 μm. For each step of the z-stack the fluorescence signal was captured (wavelengths 480/480, 595/615, 345/455). The z-stack images were deconvolved using the constrained iterative deconvolution algorithm (advanced maximum likelihood with five iterations) provided by the CellSens software. Digital images were combined with Adobe Photoshop CC 2017. All fluorescence images are shown as representative single z-plane images.

### Quantitative Real-Time PCR (qPCR)

RNA from dissected neocortices was isolated by the Trizol method, RNA from primary microglia cells were isolated by the RNeasy mini kit (Cat No./ID: 74104) and RNA was converted to cDNA as described in Babcock et al. (2006). qPCR was performed on a StepOnePlus Real-Time PCR system (Applied Biosystems) using primer and probe sequences for **HPRT** (HEX-probe AGC TTG CTG GTG AAA AGG ACC TCT CGA AGT, forward: GTT AAG CAG TAC AGC CCC AAA A-TG. Reverse: AAA TCC AAC AAA GTC TGG CCT GTA), **GAPDH** (forward: TGT CAA GCT CAT TTC CTG GTA TGA. Reverse: CTT ACT CCT TGG AGG CCA TGT AG), **CD11b** (FAM-probe TCT GTG ATG ACA ACT AGG ATC TTC-GCA GCA. Forward: CGG AAA GTA GTG AGA GAA CTG TT-TC. Reverse: ATA ATC CAA GGG ATC ACC GAA TTT), **IL-1β** (forward: CTT GGG CCT CAA AGG AAA GAA. Reverse: AAG ACA AAC CGT TTT TCC ATC TTC. Probe: AGC TGG AGA GTG TGG AT), **TNF** (forward: CCA AAT GGC CTC CCT CTC AT. Reverse: TCC TCC ACT TGG TGG TTT GC. Probe: CTC ACA CTC AGA TCA T), **Hexb** (forward: GAC TCT TTC GGG ACT TTC AC. Reverse: GTG CCA GTG AAG AAC ATT AAA C), **Clu** (Forward: GGA CAC TAG GGA TTC TGA AAT G. Reverse: AAG GGT GAG CTC TGG TTT A), **Ctsz** (forward: GAT GAG ACC TGC AAC AAC TAC. Reverse: TGG GAC CAT TGG CAT AGA), **APOE** (forward: GAG TGG CAA AGC AAC CAA. Reverse: CGT CAT AGT GTC CTC CAT CA), and **APP** (forward: CCC ACA TCG TGA TTC CTT AC. Reverse: GGG CAG CAT ACA AAC TCT AC). After normalization to HPRT and GAPDH, the average relative values for each mRNA were expressed as fold-changes compared to mRNA levels in neocortex from 3-month-old Wt (C3H/HeNxC57BL/J) mice (neocortex) or 3-month-old C57BL/6 mice (cells).

### *In situ* Hybridization (ISH)

ISH was performed on fresh-frozen sections using established procedures and previously used AP-labeled oligodeoxynucleotide probes for TNF mRNA (6 pmol/mL) and IL1β mRNA (10 pmol/mL) (Babcock et al., 2015). Following hybridization, sections were rinsed in 1x (TNF) or 2x (IL1β) saline sodium citrate (SSC) for 3 × 30 min at 55°C to melt unspecific hybridization. Then sections were rinsed in Tris-HCl buffer, pH 9.5, 2 × 10 min and developed over 72 h in AP substrates BCIP and NBT. Development was stopped by rinsing sections under running water for >1 h. Signal specificity was verified by hybridizing sections with a 100-fold excess of unlabeled probes, with hybridization buffer alone, or by hybridizing sections subsequent to treatment with RNAse. A probe specific for glyceraldehyde-3-phosphate dehydrogenase (GAPDH) mRNA (5 pmol/mL) was used to control for the quality of the tissue and the ISH procedure.

### Stereological Estimation of % Aβ Plaque Load

LPS-induced changes in Aβ plaque load were determined using unbiased stereological method as described previously (Babcock et al., 2015). Analysis was performed on an Olympus BX 50-microscope (Olympus, Germany) fitted with a U-PMTVC Japan color camera (Olympus, Germany), a Proscan Prior motorized specimen stage, and a Heidenhain MT12 microcator connected to a PC installed with the CAST-2 software (Visiopharm, Denmark). The neocortex was delineated based on naturally occurring boundaries and anatomic characteristics on sections immunostained for Aβ. Aβ plaques were defined as intensely stained brown deposit with a clear boundary compared to the background. Plaques were systematically counted in 5–10 30 μm thick sections separated by a distance of 960 μm using a point counting approach (West et al., 2009; Babcock et al., 2015). Each point hitting a plaque, represented an area (A_point_) of 6858.2 μm^2^. The percentage of neocortex covered by plaques, % Aβ plaque load, was calculated using the following equation: % Aβ plaque load = (P_tot_ × A_point_)/A_ROI_ × 100%, where A_ROI_ is the region of interest, and P_tot_ is the total number of points hitting a plaque. The coefficient of error (CE) was calculated based on the number of points counted per section and the variance of these numbers: CE = √(total variance)/P_tot_ (West et al., 1996). The CE_Group_ was calculated as follows: CE = Σ(CE^2^)/*n*, where Σ(CE^2^) is the sum of squares of the CE of the individual estimates within the group, and *n* is the number of mice in the group. The coefficient of variation, CV, was calculated as SD/Mean. For additional details on CE see West (2012).

### ELISA for Aβ_40_ and Aβ_42_

Neocortical samples were homogenized in sterile PBS including protease inhibitors (Roche Diagnostics GmbH) on ice for 6 s using an ULTRA-TURRAX Y25 basic homogenizer (Ika Werke). Samples were centrifuged 20 min at 9,000 × *g*, 4°C and supernatants were collected and stored at −80°C. Pellets were sonicated on ice for 6 s in eight volumes of 5M guanidine and 50 mM Tris-HCl and solubilized by agitation for 3 h at RT. Lastly samples were spun at 20,000 × *g* for 20 min, 4°C and supernatants containing the guanidine-soluble fraction were collected and stored at −80°C. Protein concentrations for both fractions were determined by the Bradford method (Sigma, B6916). Levels of Aβ_40_ and Aβ_42_ were measured in duplicate using commercially available ELISA kits (Thermo Fischer, KHB3481, KHB3441) following manufactures instructions using 3,3′5,5′-tetramethylbenzidine (TMB) substrate. Absorbance was measured within 30 min on a Sunrise Tecan 500 plate reader equipped with a 450 nm filter, using a reference absorbance of 650 nm.

### Statistics

Data are presented as mean ± SEM. The qPCR data were analyzed by two-way ANOVA, followed by Tukey’s *post hoc* analysis, and Aβ pathology by unpaired, two-tailed *t*-test. One outlier was removed from the qPCR dataset for TNF mRNA by Grupp’s test. Changes were considered statistically significant if *p* < 0.05, and are indicated as follows; two-way ANOVA significance level is indicated by # and *post hoc* tests and *t*-test significance levels are indicated by ^∗^. ^#/∗^*p* < 0.05, ^##/∗∗^*p* < 0.001, ^###/∗∗∗^*p* < 0.001, ^####/∗∗∗∗^*p* < 0.0001. Statistical analysis was performed by Prism 6.0 software (GraphPad Software).

## Results

### Repeated LPS Administration Increases TNF mRNA Levels in Both Wt and Tg Mice

**FIGURE 1:**
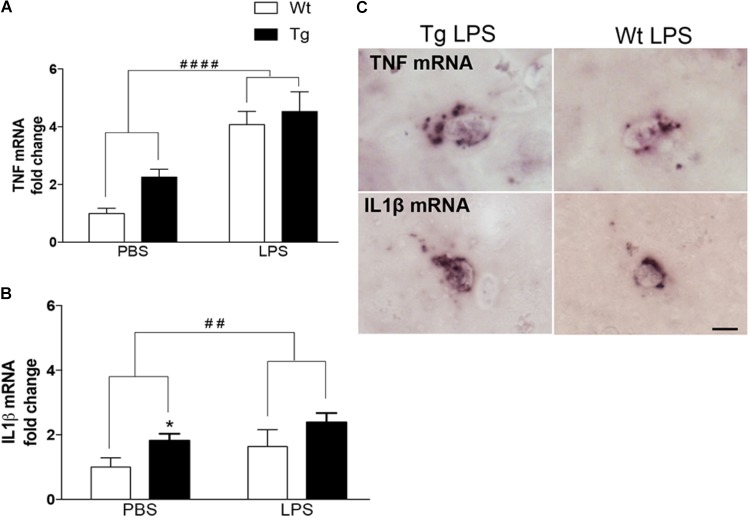
Repeated systemic LPS administration increases cortical TNF and IL1β mRNA levels. **(A,B)** Quantitative PCR analysis showing significantly elevated TNF and IL1β mRNA levels after LPS administration in Wt and Tg mice. Data was analyzed by two-way ANOVA with LPS administration and genotypes as variables (significance level indicated by #) followed by Tukey’s test (significance level indicated by ^∗^). Bars and error bars represent mean ± SEM. ^∗^*p* < 0.05, ^##^/^∗∗^*p* < 0.01, ^####^/^∗∗∗∗^*p* < 0.0001. **(C)** *In situ* hybridization showing representative TNF and IL1β mRNA^+^ cells in the neocortex of LPS-injected Wt and Tg mice. Scale bars = 10 μm.

We have previously shown that microglia produce TNF and IL1β in 9-month-old Tg mice (Babcock et al., 2015) and LPS is known as a strong inducer of microglial cytokine production (Perry et al., 2007). In line with this, the TNF mRNA levels in neocortex were significantly influenced by the LPS administration [F_(1,36)_ = 38.6, p < 0.0001, two-way ANOVA], giving rise to a twofold to fourfold increase in Wt [p < 0.0001, Tukey’s] and Tg [p < 0.05] mice (Figure 1A). There was also an effect of genotype [F_(1,36)_ = 4.5, p < 0.05], however, no differences within individual PBS and LPS groups (Figure 1A). Similarly, IL1β mRNA levels were influenced both by the LPS administration [F_(1,37)_ = 9.8, p < 0.01] and the genotype [F_(1,37)_ = 16.6, p < 0.0001], and with significantly higher IL1β mRNA levels in PBS-injected Tg versus Wt mice [p < 0.05, Tukey’s] (Figure 1B). ISH showed scattered TNF mRNA^+^ and IL1β mRNA^+^ cells with a parenchymal location in the neocortex in all groups of mice (Figure 1C), similar to descriptions in Babcock et al. (2015).

### Repeated LPS Administration Ameliorates Aβ Pathology in Tg Mice

**FIGURE 2:**
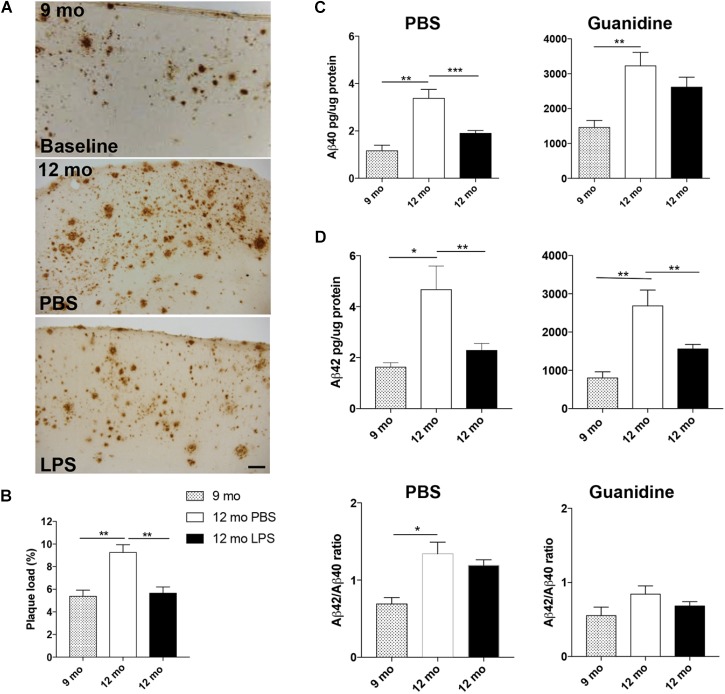
Repeated systemic LPS administration ameliorates neocortical Aβ pathology. **(A,B)** Plaque load estimation of 6E10-stained Aβ plaques in neocortex **(A)**, at 9 months (baseline) (top panel) and after 13 weeks of LPS/PBS administration in Ncx (bottom panels of **A**) shows reduced neocortical plaque load at 12 months of age in LPS-injected Tg mice **(B)**. **(C)** ELISA of PBS and guanidine fractions of Aβ_40_ and Aβ_42_ levels in neocortex show a reduction of the levels of PBS-soluble Aβ_40_ and Aβ_42_ after LPS administration and of guanidine-soluble Aβ_42_. **(D)** Aβ_40_/Aβ_42_ ratios are unaffected by the administration. Unpaired, two-tailed *t*-test, *n* = 6–10/group. Bars and error bars represent mean ± SEM. ^∗^*p* < 0.05, ^∗∗^*p* < 0.01, ^∗∗∗^*p* < 0.001. Scale bar: 50 μm.

To investigate whether LPS administration influences the Aβ pathology in the neocortex, plaque load was estimated in Tg mice injected with LPS or PBS from 9 to 12 months of age. In addition, the plaque load was estimated at baseline in 9-month-old Tg mice, allowing us to observe an increase with age in PBS-injected Tg mice [*p* < 0.01, unpaired, two-tailed *t*-test] (Figures 2A,B and Table 2). In comparison, plaque load was significantly lower in the neocortex in LPS- versus PBS-injected mice at 12 months of age [*p* < 0.01] (Figure 2B and Table 2), resulting in a 39% reduction in the plaque load in the LPS-injected mice, thereby being comparable to baseline (Figure 2B and Table 2). The levels of Aβ_40_ and Aβ_42_ were quantified in contralateral neocortex samples and both PBS- and guanidine-soluble fractions were evaluated. As expected, there was a significant age-dependent increase of Aβ_40_ and Aβ_42_ in both fractions from 9 to 12 months [*p* < 0.01 or *p* < 0.001] (Figure 2C). Importantly, a significant reduction was observed in the PBS fraction of both Aβ_42_ and Aβ_40_ [*p* < 0.01 or *p* < 0.001, respectively; *n* = 6 (PBS) and *n* = 11 (LPS)], and of Aβ_42_ [*p* < 0.01], but, however, not Aβ_40_, in the guanidine fraction of LPS- versus PBS administration at 12 months of age (Figure 2C). The ratio of Aβ_42_/Aβ_40_ was not influenced by the LPS administration in neither the PBS nor the guanidine fraction (Figure 2D). Thus, the repeated systemic LPS administration mitigated the normally occurring age-dependent increase in Aβ pathology in the neocortex of 12-month-old female Tg mice.

**Table 2:** % Aβ plaque load in neocortex.

Group	Baseline	PBS	LPS
Aβ load (%)	5.38	9.25	5.65
CV (SD/mean)	0.13	0.26	0.21
CE_group_ (SEM/mean)	0.11	0.10	0.13
*n*	4	8	8

*CE_group_, average coefficient of error, CV, coefficient of variation (=SD/mean).*

### Microglia Cluster Around Aβ Plaques in Both LPS- and PBS-Injected Tg Mice

**FIGURE 3:**
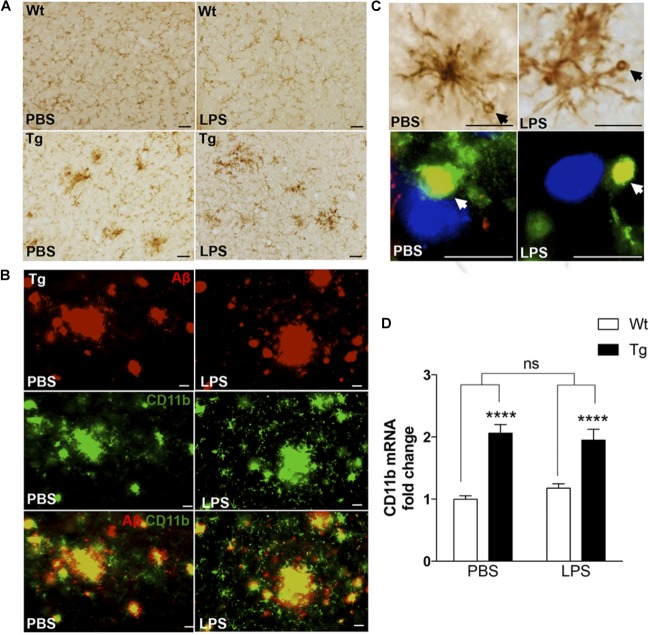
CD11b^+^ cells clustering around Aβ plaques display Aβ-containing vacuole-like structures. **(A)** IHC staining showing CD11b^+^ cells in the neocortex of PBS- and LPS-injected Tg and Wt mice. Cells with altered morphology and with some forming clusters are more abundant in Tg mice compared to Wt mice. **(B)** Double immunofluorescence staining showing co-localization (yellow) of 6E10^+^ Aβ plaques (red) and CD11b^+^ cells (green) in both PBS- and LPS-injected Tg mice. **(C)** CD11b^+^ cells with vacuole-like structures are observed in both PBS- and LPS-injected Tg mice, and double immunofluorescence shows some vacuoles to be Aβ^+^ (yellow). **(D)** Quantitative PCR analysis showing a significant effect of genotype, and no effect of LPS administration, on the CD11b mRNA levels. Two-way ANOVA followed by Tukey’s multiple comparisons test. Bars and error bars represent mean ± SEM, *n* = 7–10 per group, ^∗∗∗∗^*p* < 0.0001. Scale bars: 25 μm **(A)**, 20 μm **(B)**, 50 μm (**C**, top), and 10 μm (bottom).

As microglia can take up, sequester, and clear Aβ from the neuropil (Koenigsknecht and Landreth, 2004), we next evaluated microglial response to Aβ in LPS- and PBS-injected mice by IHC staining for the microglial surface β-integrin, CD11b. Compared to the CD11b^+^ microglia in Wt mice, which had a round to elongated soma with finely ramified processes (Figure 3A), many CD11b^+^ microglia in Tg mice had hypertrophic cell bodies and processes, characteristic of activated microglia. Furthermore, the microglia frequently formed clusters (Figure 3A), consistent with microglia aggregating around deposits of Aβ. This was confirmed by double-IF staining for CD11b and Aβ (Figure 3B), which additionally showed that the vacuole-like structures observed in CD11b^+^ processes were filled with Aβ^+^ material in both LPS- and PBS-injected Tg mice (Figure 3C). The CD11b^+^ vacuole-like structures were not observed in Wt mice. In line with this, there was a statistically significant, approximately twofold, genotype-dependent up-regulation of CD11b mRNA levels [*F*_(1,37)_ = 66.7, *p* < 0.0001, two-way ANOVA], in the neocortex of both PBS- and LPS-injected Tg mice [*p* < 0.0001, Tukey’s, both comparisons], and with no effect of LPS on CD11b mRNA levels in neither Tg nor Wt mice [*F*_(1,37)_ = 0.08.6, *p* = 0.77] (Figure 3D).

### Genotype and LPS Impact Pathways of Neuroinflammation, Amyloidosis, and Cellular Stress

In order to elucidate the downstream effects of repeated LPS administration in the Tg mouse, we analyzed the changes in protein expression in hippocampal samples from the LPS- and PBS-treated Tg and Wt mice by a global quantitative proteomics approach. This was done by iTRAQ-8plex labeling and off-line fractionation before LC-MS/MS analysis (Supplementary Figure S1A). More than 2,600 proteins were quantified with at least two unique peptides (Table 1 and Supplementary Table S3).

Initially, the differences in the hippocampal proteome between Tg and Wt mice were determined. Twenty-four proteins were significantly differently regulated in Tg and Wt proteomes (Table 3). These proteins included, besides the expected increased expression of APP in Tg mice, an increase in additional proteins known to be involved in AD, such as glial fibrillary acidic protein (GFAP), APOE, Clu, vimentin (VIM) and integral membrane protein 2B (ITM2b) (Table 3).

**Table 3:** Significantly regulated proteins in hippocampal tissue from PBS- and LPS-injected Wt and Tg mice (limma test with *q* < 0.01).

Gene name	Acc. no.	Tg PBS vs. Wt PBS	Tg LPS vs. Wt LPS	Tg LPS vs. Tg PBS
Gfap	P03995	1.79	1.63	
H3f3c	P02301		1.30	
Apoe	P08226	1.87	1.51	
Clu	Q06890	1.60	1.59	
Vim	P20152		1.41	
Hexb	P20060		1.22	
Dctn3	Q9Z0Y1		1.16	
Grm2	Q14BI2		0.86	
calb2	Q08331		1.20	
App	P12023	1.69	1.79	
Neo	P97798		1.18	
Cnn3	Q9DAW9		1.16	
Ctsz	Q9WUU7	1.35	1.30	
Col6a1	Q04857		1.24	
Itm2b	O89051		1.17	
Osbpl8	B9EJ86		0.76	
Htra1	Q9R118		1.23	
Sncb	Q91ZZ3	0.84		
Dock9	Q8BIK4	1.19		
Atp2b4	Q6Q477	0.73		
Hist1h1b	P43276	0.80		
C4b	P01029	1.63		0.83
Apbb1	Q9QXJ1	0.82		
Ndufab1	Q9CR21			1.14
Eef1b	O70251			1.15
Rpl21	O09167			1.17
Tardbp	Q921F2			1.11
Ptpr	Q64455			0.85
Arhgap23	Q69ZH9			0.83
Tspan2	Q922J6			0.77
Hdgf	P51859			1.15
Hapln4	Q80WM4			0.86
D17Wsu104e	Q9CPT4			1.11
F8a1	Q00558			0.85
Gsr	P47791			0.82
Timm10b	Q9WV96			0.80
Cep97	Q9CZ62			0.83
Osbpl6	Q8BXR9			0.81
Rsu1	Q01730			1.14
Mrpl4	Q9DCU6			0.87
Xrn1	P97789			0.78

The regulated proteins were analyzed using the IPA software with focus on disease pathways. Regulated proteins were significantly enriched in pathways of neuroinflammation and amyloidosis (Supplementary Figure S2A). Proteins were also analyzed for their involvement in biological processes, where retinoic receptor activation and the complement system were significantly different with LPS administration (Supplementary Figure S2B). Furthermore, pathways related to reelin signaling in neurons, production of reactive oxygen (RO) and reactive nitrogen (NO) species, clathrin-mediated endocytosis signaling, signaling by RHO GTPases, 14-3-3 signaling and amyloid processing were altered between genotypes (Supplementary Figure S2B).

The expression levels of 19 proteins were significantly changed in response to the LPS administration in the Tg mice, while no proteins were found changed in LPS-injected Wt mice (Tables 1, 3). When clustering significantly changed proteins into pathways in Tg mice using IPA, three pathways were enriched, namely retinoid receptor activation, redox reactions and the complement pathway (Supplementary Figure S2C).

### Genotype Impacts the CNS Myeloid Cell Proteome

The CNS myeloid cell proteome of Tg mice was defined in CD11b^+^ cells sorted from cerebral cortex homogenates, followed by an iTRAQ-4plex protocol with off-line fractionation (Supplementary Figure S1B). We were able to quantify 467 proteins with at least two unique peptides (Table 1 and Supplementary Table S4). Among these, proteins translated from what has been considered microglial-specific genes were identified including *hexb, crybb1, fcrls, c1qa, itgam*, and *aif1* (Crotti and Ransohoff, 2016). From these, 52 proteins were considered regulated with a protein ratio below 0.7 or above 1.3 (Table 4). The proteins that showed the largest increase in Tg CD11b^+^ cells included known AD-associated proteins, such as APOE, APP, and Clu. Furthermore, the lysosomal protein Ctsz was significantly increased in Tg CD11b^+^ cells.

**Table 4:** Proteins regulated between Tg CD11b^+^ cell and Wt CD11b^+^ cell proteomes.

Acc. no.	Gene name	Up-regulated	Acc. no.	Gene name	Down-regulated
Q3TXU4	Apoe	5.608	Q921M7	Fam49b	0.696
P12023	App	4.351	Q3U1J4	Ddb1	0.688
Q545I6	Ctsz	2.174	B2RVP5	H2afv	0.68
Q549A5	Clu	1.865	Q08879	Fbln1	0.679
P48036	Anxa5	1.828	Q4FZE6	Rps7	0.677
P29351	Ptpn6	1.552	B2RWH3	Hist2h2aa1	0.676
Q4KML7	Ezr	1.511	P63038	Hspd1	0.674
E9Q616	Ahnak	1.506	Q61599	Arhgdib	0.672
P24452	Capg	1.48	Q9R112	Sqor	0.671
P48678	Lmna	1.445	P20918	Plg	0.667
O54734	Ddost	1.391	Q543J5	Serpinc1	0.662
Q8R2Q0	Trim29	1.382	P60879	Snap25	0.661
Q91WJ8	Fubp1	1.379	Q4FK74	Atp5d	0.652
Q9D964	Gatm	1.366	Q8BG05	Hnrnpa3	0.643
E9Q557	Dsp	1.34	P26350	Ptma	0.638
O08585	Clta	1.318	P47877	Igfbp2	0.637
P17879	Hspa1b	1.308	Q6GQT1	A2m	0.634
P50516	Atp6v1a	1.307	Q9EQU5	Set	0.633
Q9QXS1	Plec	1.307	P01942	Hba	0.633
Q99KK2	Cmas	1.302	P62307	Snrpf	0.623
			P21460	Cst3	0.608
			Q9Z0P5	Twf2	0.587
			Q60668	Hnrnpd	0.585
			Q50HX4	Rab14	0.58
			P10107	Anxa1	0.568
			Q9D2V7	Coro7	0.552
			Q53X15	S100a8	0.549
			P51437	Camp	0.54
			P10853	Hist1h2bf	0.539
			Q9R0G6	Comp	0.539
			Q921I1	Tf	0.378
			P84244	H3f3a	0.266

*Proteins were considered up-regulated when the ratio was >1.3 and down-regulated when the ratio was <0.7.*

**FIGURE 4:**
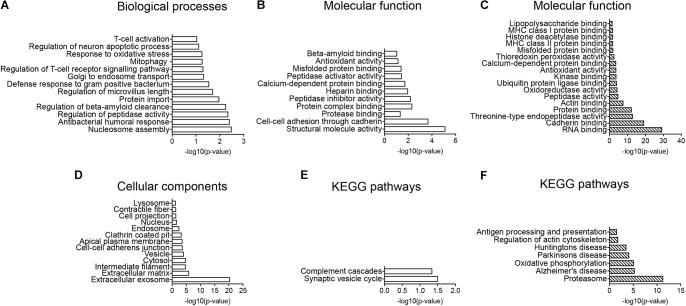
GO-term and enrichment of KEGG pathways analysis. **(A,B,D,E)** GO-term analysis of protein pathways enriched in the CD11b^+^ cell proteome of Tg versus Wt **(A,B,D)** and enrichment of KEGG pathways **(E)**. **(A)** Enrichment of biological processes. **(B)** Enrichment of molecular function. **(D)** Enrichment of cellular components. **(C,F)** GO-term enrichment analysis of molecular functions **(C)**, and enrichment of KEGG pathways **(F)** in potential CD11b^+^ cell proteins in hippocampus. Data was analyzed by the DAVID software.

Regulated proteins were analyzed using the enrichment of GO-terms showing biological processes evolving around Aβ clearance mechanisms and oxidative stress including “regulation of beta-amyloid clearance, Golgi to endosome transport, protein import, mitophagy, response to oxidative stress and regulation of intrinsic apoptotic signaling pathway” (Figure 4A). The enriched molecular function of proteins, supported regulated proteins to be involved in clearance with GO-terms including “protease binding, protein complex binding, peptidase activator activity, misfolded protein binding and beta-amyloid binding” (Figure 4B). The cellular component analysis showed enrichment of lysosomal, endosomal, clathrin-coated pit, cell projections and vesicle proteins (Figure 4D). KEGG pathway enrichment indicated CD11b^+^ cells to be more activated in Tg mice with the complement pathway being significantly enriched in the dataset (Figure 4E).

### Overlap of the Total CNS Myeloid Cell and Total Hippocampal Proteomes

The total CD11b^+^ cell and the total hippocampal proteomes were matched to extract proteins that were potential microglial proteins affected by LPS administration, resulting in a list of 291 proteins (Supplementary Table S4). Performing GO-term analysis on the shared proteins, the enrichment of molecular function indicated immune response molecules to be enriched including “LPS binding proteins, MHC class I and II protein binding, misfolded protein binding” as well as oxidative stress proteins (Figure 4C). Furthermore, the enrichment of KEGG pathways indicated proteasome, AD, oxidative phosphorylation, cytoskeleton as well as antigen processing and presentation pathways to be enriched (Figure 4F).

### The CNS Myeloid Cell Proteome Overlaps With the Hippocampal Proteome Impacted by Genotype and LPS Administration

**FIGURE 5:**
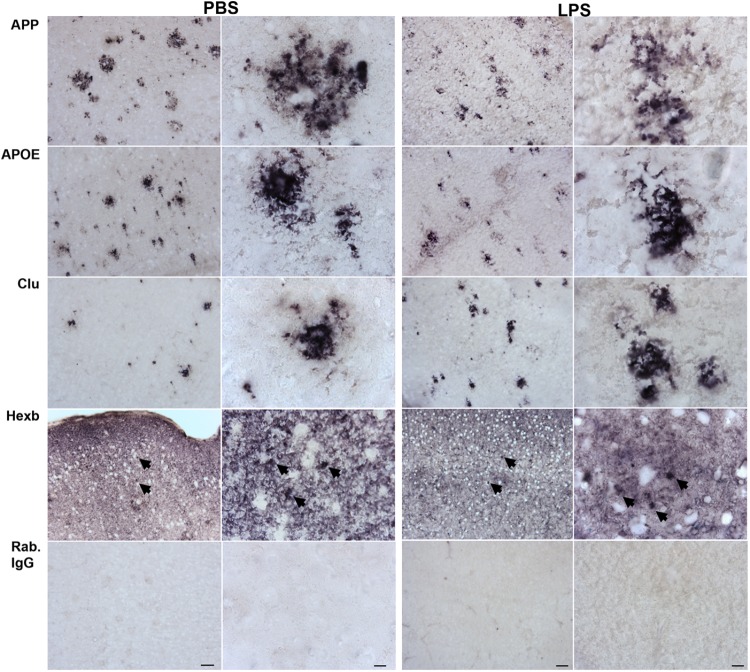
APP, APOE, Clu, and Hexb protein expression in neocortex of Tg mice. Sections from Tg mice injected with LPS or PBS (*n* > 2/group, two rounds of staining) were immunohistochemically stained using primary rabbit-antibodies and using an alkaline phosphatase conjugated secondary antibody yielding a bluish-black reaction product. The staining for APP, APOE, and Clu showed an Aβ-plaque-like distribution pattern. The Hexb staining displayed both a diffuse staining and a punctate staining of subcellular structures (arrows). IgG controls showed no staining. Scale bars: 50 μm (low power), 10 μm (high power).

**FIGURE 6:**
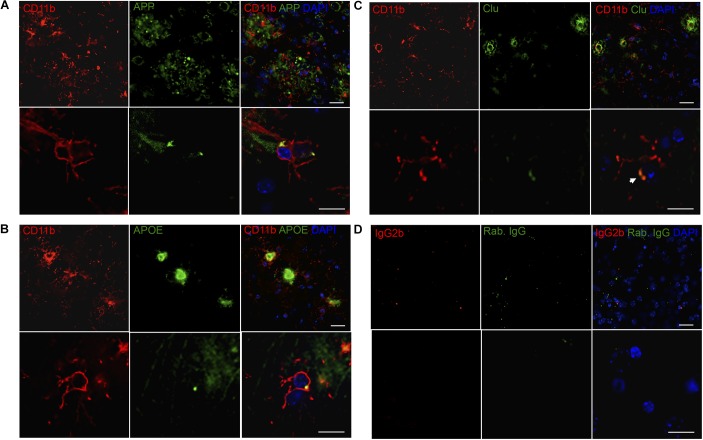
APP, APOE, and Clu show variable co-localization to CD11b^+^ cells in neocortex in Tg mice. **(A–C)** Double immunofluorescence staining was performed for CD11b and APP **(A)**, APOE **(B)**, and Clu **(C)** on sections from LPS-injected Tg mice (*n* > 2/group, two rounds of staining). Images represent a deconvolved single z focus plane. Microglia (red) and APP, APOE and Clu (green). Sections were counterstained with the nuclear marker DAPI (blue). **(D)** Rat IgG2b and rabbit IgG controls showed no specific staining. Scale bars: 20 μm (low power) and 10 μm (high power).

When comparing the proteins found regulated in hippocampi from PBS- and LPS-injected Tg mice to the total CD11b^+^ cell proteome, four proteins were shared including the lysosomal cysteine protease Ctsz, APP, APOE and Clu. We initially validated the expression of all four proteins as well as the microglial-expressed HexB, in tissues from LPS- and PBS-injected Tg mice by IHC staining (Figure 5). The staining pattern of APOE, APP, and Clu showed an Aβ-plaque-like distribution (Figure 5), not observed in the Wt mice (Supplementary Figure S3). The staining for Ctsz was suboptimal and is not shown. Hexb showed a more diffuse distribution, however, with an additional punctate staining (arrows in Figure 5). Double-IF staining was performed on Tg brain sections to test for co-localization of APP, APOE, Clu as well as microglial marker Hexb to CD11b^+^ cells (Figure 6). The IF-signal for APOE and Clu were both co-localized to CD11b^+^ cells showing an Aβ-plaque-like distribution (Figures 6B,C), while this was rarely observed for APP (Figure 6A). The double-IF staining for Hexb and CD11b was suboptimal (data not shown). Control stainings with rabbit IgG were either devoid of staining (Figure 5), or in case of the double-IF stainings contained minimal punctate staining (Figure 6D). Orthogonal views are shown in Supplementary Figure S6.

**FIGURE 7:**
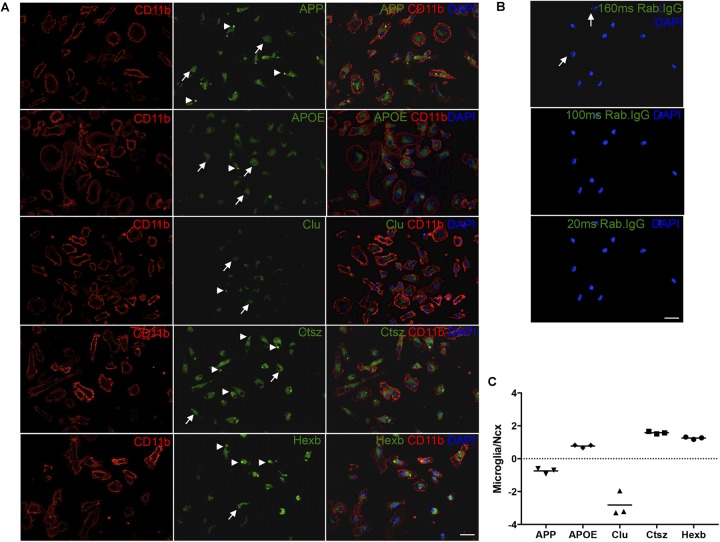
APP, APOE, Clu, Ctsz, and Hexb protein and mRNA expression in primary microglia. **(A,B)** Double-IF showing protein expression of APP, APOE, Clu, Ctsz, and Hexb in primary microglia from newborn C57BL/6 mice **(A)**, also showing the rabbit IgG controls **(B)**. White arrows in **(A)** indicate diffuse staining, white arrow heads indicate punctate staining **(A)**. Rabbit IgG controls in **(B)**, captured with the same exposure time as the primary antibody, showed no noticeable signal. Top panel: Rab. IgG for Ctsz, 160 ms. Middle panel: Rab. IgG for Hexb, APP, APOE, 100 ms. Bottom panel: Rab. IgG for Clu, 20 ms. Images represent a deconvolved single z focus plane. Scale bars: 20 μm **(A,B)**. **(C)** APP, APOE, Clu, Ctsz, and Hexb mRNA levels in primary microglia relative to neocortex samples from C57BL/6 mice. Each data point represent the average of triplicates of triplicates and are presented on a log_10_ scale for comparison to whole neocortex tissue. Above ‘0’ shows that the gene is more abundantly expressed in primary microglia than in whole Ncx tissue, below ‘0’ shows that the gene is less abundantly expressed in primary microglia than in whole neocortex tissue.

Next, to additionally validate the expression of Ctsz, APP, Clu, and APOE in microglia, double-IF was performed on primary microglia isolated from newborn C57BL/6 mice with inclusion of HexB, which is known to be expressed in microglia (Crotti and Ransohoff, 2016) as a control. Ctsz, APP, Clu, APOE, and Hexb were all expressed in CD11b^+^ primary microglia (Figure 7A). The stainings showed a diffuse intracellular signal (white arrows) with additional punctate signal (white arrowheads). Staining in which the primary antibody was substituted with inert rabbit IgG was devoid of specific-like signal (Figure 7B), lending support of the results obtained on the tissue sections (Figure 6). Orthogonal views can be seen in Supplementary Figure S7. Additionally, the ability of microglia to transcribe these genes was examined by qPCR. The mRNA levels of APP, APOE, Clu, Ctsz, and Hexb were determined relative to the expression level of these genes in the neocortex of 3-month-old C57BL/6 mice (Figure 7C). The qPCR results showed that APOE, Ctsz, and Hexb was expressed in higher levels in primary microglia relative to neocortex, whereas APP and Clu mRNA could be detected in primary microglia, but their expression was lower than in neocortex (Figure 7C).

### CNS Myeloid Proteins Affected by Genotype and LPS Administration Are Expressed in Postmortem Neocortex Tissue From AD Cases

**FIGURE 8:**
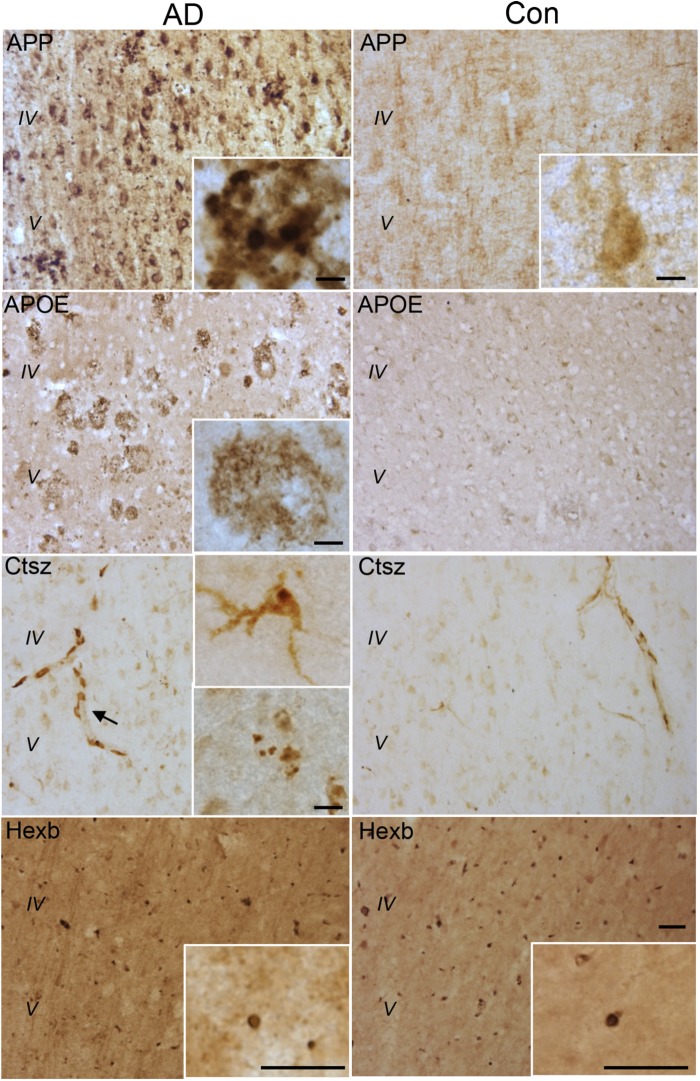
APP, APOE, Ctsz, and Hexb protein expression in Ncx of human post-mortem AD and control (Con) cases. For immunohistochemistry was used vibratomic sections from five AD and three control cases. The stainings were performed using primary rabbit-antibodies and the Envision system yielding a brown reaction product. The APP staining showed neuronal localization as well as accumulation in amyloid plaque-like structures (insert) in AD cases. The APOE staining showed an amyloid plaque-like distribution in AD cases. The Ctsz staining was localized to perivascular cells in AD and control cases (arrow). In AD cases the Ctsz staining additionally occurred as a diffuse and punctate staining in cells with a microglial-like morphology (insert, top) and as aggregates of punctate staining (insert, bottom). The Hexb staining showed a punctate staining of subcellular structures in AD and control cases, however with high background staining. IgG controls showed no staining (See Supplementary Figure S4). Scale bars: 50 μm (low power) and 10 μm (inserts).

**FIGURE 9:**
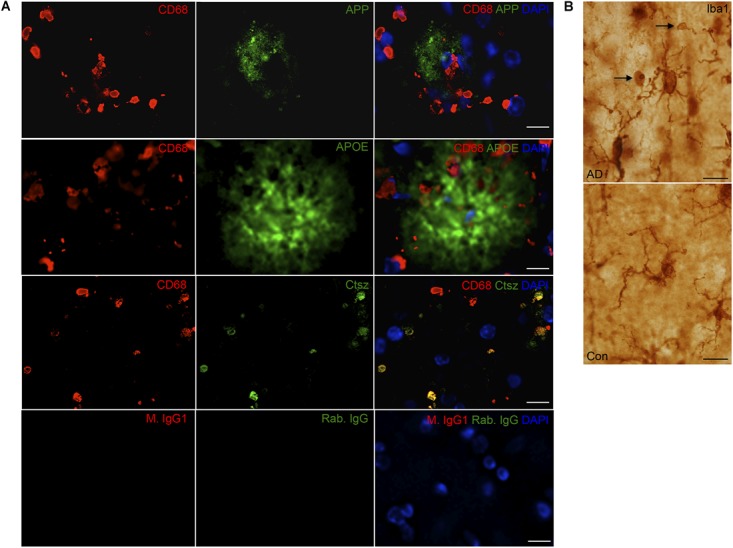
**(A)** APP, APOE, and Ctsz show variable co-localization to CD68^+^ cells in neocortex of human AD cases. Double immunofluorescence staining was performed on human sections (*n* > 2/group, two rounds of staining). CD68 (red) and APP, APOE, Ctsz (green), sections were counterstained with the nuclear marker DAPI. Images represent a deconvolved single z focus plane. Co-expression was observed for Ctsz and the lysosomal marker CD68. IgG control showed no staining. **(B)** Iba1 staining of AD and control cases showed Iba1^+^ microglial cells with vacuolar structures along their processes of up to 4–5 μm in diameter [figure modified from Babcock and Finsen (2012) with permission from the Carlsberg Foundation]. Scale bars: 20 μm (APP, APOE in **A**), 10 μm (Ctsz, IgG in **A, B**).

The expression of the CNS myeloid cell proteins regulated in Tg mice, APP, APOE, Ctsz as well as Hexb, was also evaluated in neocortex tissue from five AD cases and three cognitively healthy controls (Supplementary Table S1). Supplementary Figures S4A,B shows group-representative images of IHC stainings for Aβ, pTau and Iba1 from one AD and one control brain. APP, APOE, and Ctsz all showed an altered pattern of immunoreactivity in AD compared to controls (Figure 8 and Supplementary Figure S4C). APP immunoreactivity in AD cases, showed both neuronal expression (Supplementary Figure S4C) as well as being located to aggregates (Figure 8) resembling Aβ plaques in parallel sections (Supplementary Figures S4A–C), whereas controls only showed a faint expression in neurons. The distribution of APOE (Figure 8) was comparable to that of Aβ (Supplementary Figure S4), with no clear signal in the control cases (Figure 8). Double-IF showed that APP and APOE co-localized with CD68^+^ cells, but due to CD68 being a lysosomal marker, co-expression of these proteins could not be inferred (Figure 9A). For orthogonal views, see Supplementary Figure S8.

Immunoreactivity for Ctsz was observed in perivascular cells in both AD and control brains, and in aggregates of punctate staining as well as in cells with a microglial-like morphology, some of them also showing a punctate staining in AD, but not in control brains (Figure 8A and Supplementary Figure S4C). None of this staining was observed in the substitution controls (Supplementary Figure S5). Double-IF for Ctsz and CD68 showed co-expression of Ctsz in CD68^+^ lysosomal structures of sizes up to 4–5 μm (Figure 9A), thereby being of the same size as the large vacuole-like structures observed in the processes of some of Iba1^+^ microglial cells in some of the AD brains (Figure 9B). The double-IF staining did not allow identification of the faintly Ctsz-immunoreactive microglial-like cells observed in the chromogenic staining (Figure 8), likely due to autofluorescence of the human brain tissue. The Hexb protein showed a punctate staining pattern, staining both smaller and larger subcellular structures in brain from both AD and control cases (Figure 8 and Supplementary Figure S4C).

## Discussion

### Main Findings

The results of this study showed that repeated systemic administration of LPS increased cortical levels of TNF mRNA and IL1β mRNA in both Tg and Wt mice, demonstrating that systemically administered LPS impacted neuroinflammation in the neocortex. The repeated systemic LPS administration led to a significant 39% reduction in cortical Aβ plaque load in the Tg mice at 12 months of age supported by overall lower levels of Aβ_40_ and Aβ_42_ in the contralateral neocortex samples. To our knowledge, this is the first comprehensive study, which has investigated the effect of repeated systemic LPS administration on Aβ pathology in APP_swe_/PS1_ΔE9_ mice.

Using proteomics, LPS was found to influence hippocampal protein pathways of the complement system, oxidative stress response and the activation state of retinoid receptors. In addition, proteome comparison of CNS myeloid cells isolated from Tg and Wt mice at 24 months of age showed that also Tg CD11b^+^ cells were enriched in protein pathways of the complement system, microglial clearance mechanisms and oxidative stress response. This indicates that CNS myeloid cells are influenced by the accumulation of Aβ plaques in Tg mice inducing a state of chronic increased activity, affecting functional aspects of CNS myeloid cells and thereby microglial function, that could impact disease severity.

Lastly we found four proteins APP, APOE, Clu, and Ctsz to be regulated with genotype both in hippocampus and in CNS myeloid cells as well as showing an altered immunoreactivity pattern in post-mortem cortex tissue from AD cases relative to cognitively healthy controls. Especially the Ctsz protein was of interest due to its presence in perivascular cells as well as microglial-like cells and CD68^+^ lysosomes. This is, to our knowledge, a novel observation of Ctsz expression in post-mortem cortex tissue from AD cases.

### What Is Already Known in the Field?

Previous research has reached consensus that systemically administrated LPS crosses the blood brain-barrier (BBB) in APP_swe_/PS1_ΔE9_ mice, especially in older mice (Cunningham, 2013; Minogue et al., 2014), but whether or not LPS crosses the intact BBB in a Wt mouse is still being debated. Under all circumstances, LPS that enters the CNS will be able to act on microglia by binding to LPS-binding receptors, especially Toll-like receptor 4 (TLR4)/CD14 and TLR2 (Liu et al., 2005; Doens and Fernández, 2014). This can lead to an increased expression of pro-inflammatory cytokines, such as TNF and IL1β, and increased microglial expression of Aβ-binding receptors as well as LPS-binding receptors, which can also bind Aβ (Doens and Fernández, 2014). The parenchymal localization of the TNF mRNA^+^ and IL1β mRNA^+^ cells in all groups of mice, our former demonstration of increased TNF and IL1β protein expression in a subset of microglia in both Tg and Wt mice (Babcock et al., 2015), and literature on LPS induction of TNF and IL1β in microglia (Cunningham, 2013), indicate disease associated microglia as an important source of the elevated TNF mRNA and IL1β mRNA levels in both Tg and Wt mice after LPS administration. While the localization of the TNF mRNA^+^ and IL1β mRNA^+^ cells in principle exclude perivascular cells as sources of the increased levels of TNF mRNA^+^ and IL1β mRNA^+^, another source of CNS myeloid cells could be immigrating monocyte-derived macrophages, which can be found in 12-month-old Tg mice (Babcock et al., 2015). There is no doubt, however, that subsets of microglia in both APP_swe_/PS1_ΔE9_ and littermate Wt mice synthesize both TNF and IL1β, as well as take up endogenously produced Aβ, and also that microglial production of TNF and IL1β appears to be inversely correlated to their content of Aβ (Babcock et al., 2015).

Studies on the effect of LPS on Aβ-plaque load have reached mixed conclusions, likely due to differences in study designs. However, a 44% reduction in cortical Aβ plaque load 7 days after the last of two i.p. injections with LPS (0111:B4, 0.5 mg/kg) given with 1 month interval was previously reported for 14-month-old APP_swe_ (Tg2576) mice (Quinn et al., 2003). In comparison, cortical Aβ40 and Aβ42 levels, as well as APP levels, increased threefold in approx. 1-year-old APP_swe_ mice injected i.p. with LPS (055:B5, 0.5 mg/kg) given once weekly for 12 weeks (Sheng et al., 2003). A third study in which LPS (055:B5, 0.5 mg/kg) was administered i.p. to 4-month-old 3xTg-AD mice twice weekly for 6 weeks, and sacrificed 24 h after last injection, reported no changes in protein levels of either soluble or non-soluble Aβ_40_ and Aβ_42_ levels in whole-brain preparations (Kitazawa, 2005). The observation of a significant reduction of Aβ pathology in the 12-month-old APP_swe_/PS1_ΔE9_ mice, with the Aβ plaque load and Aβ_40_ and Aβ_42_ levels being reduced toward baseline levels, might be important for the discovery of pathways and new molecular targets with the potential to lower Aβ levels at an early stage of disease.

There are several possible explanations for the mitigating effect of the repeated systemic LPS administration on Aβ pathology; (1) increased clearance, likely reflecting that the microglia become more effective in clearing Aβ from the neural parenchyma (Mandrekar et al., 2009), (2) reducing the production of APP and/or shifting the processing of APP into the non-amyloidogenic direction (Lee et al., 2008), (3) a combination of these two options, and (4) other explanations, not discussed in this study. There are also several plausible reasons for why microglia could be more effective in clearing Aβ from the neuropil upon LPS administration, such as the effect of LPS on microglial TLR4/CD14 and TLR2. In addition, increased uptake of soluble Aβ by microglial macropinocytosis could be a reason as suggested by some studies (Mandrekar et al., 2009). In line with findings by others in mouse models of AD (Simard et al., 2006; Frank et al., 2008; Hickman et al., 2008) and observations in the postmortem cortex from AD cases (Vehmas et al., 2003; Liu et al., 2005; Bradshaw et al., 2013), we found that the CD11b^+^ microglia in LPS- and PBS-injected 12-month-old Tg mice clustered around the Aβ plaques and appeared to sequester Aβ in vacuole-like structures, in their processes. These structures were reminiscent of phagocytic pouches previously shown to contain lysosomes (Sierra et al., 2010; Liaury et al., 2012), and with our observations of Ctsz^+^CD68^+^ lysosomal and Iba1^+^ vacuole-like structures in AD brains, and in line with our former flow cytometry demonstration of CD11b^+^ (CD45^+^) microglia being loaded with endogenously produced Aβ in Tg but not Wt mice (Babcock et al., 2015). These data taken together, suggest that microglia in Tg mice had the capacity to ingest Aβ, thereby also raising the possibility that the microglial capacity to clear Aβ might be enhanced in the LPS-injected mice, although this was not reflected in an overlap between the proteome of LPS- versus PBS-injected Tg mice and the CNS myeloid cell proteome.

### How Does This Study Add to Existing Knowledge?

The proteome analysis of hippocampal tissue from Wt and Tg mice obtained in this study, was supported by its overlap in regulated proteins (amongst others APP, APOE, and Clu) with a previous proteomics study performed on this Tg model (Kempf et al., 2016). In the present study, the proteome analysis showed that the major genotype-associated differences in addition to amyloidogenesis and inflammation included the complement system and the oxidative stress response, pathways that were also regulated by LPS and that have previously been suggested to affect AD pathogenesis (Sultana and Butterfield, 2010; Shen et al., 2013). Interestingly, the ITM2b protein, also called BRI2, was linked to the amyloidosis pathway and increased in Tg mice. This protein has been shown to be able to interact with APP (Matsuda et al., 2005), and thereby block the APP cleavage sites of the secretases responsible for Aβ production, thus hindering the formation of Aβ peptides (Matsuda et al., 2011a). In addition, mutations in the BRI2 gene is known to cause familial Danish and British dementia, two types of dementia that, like AD, are characterized by a deposition of Aβ and formation of neurofibrillary tangles (Matsuda et al., 2011b). Accumulation of ITM2b has been observed around amyloid plaques in neocortex and hippocampus of human AD cases (Garringer et al., 2017), suggesting a shared mechanism between familial Danish/British dementia and AD, centered on amyloidosis. In comparison, LPS especially affected the retinoid receptor activation state which is known to be involved in LPS-induced inflammatory responses in macrophages of the periphery, suggesting that LPS might impact on microglial clearance of Aβ via retinoid receptor activation (Núñez et al., 2010; Wang et al., 2015). Still, in the present analysis no single protein was shared between the CNS myeloid cell proteome and the proteins that were differently regulated due to LPS administration in Tg mice, suggesting that the effect of LPS might be more complex.

The four proteins, APP, APOE, Clu, and Ctsz were regulated both in hippocampal tissue from Tg mice and CNS myeloid cells isolated from Tg mice. In addition we observed an altered immunoreactivity pattern in cortical tissue from Tg versus Wt mice and in post-mortem cortex from AD cases versus cognitively healthy controls of these proteins, however, with the limitation that we failed to obtain IHC results for Ctsz on the mouse tissue and for Clu on the human tissue. The APP protein is primarily a neuronal membrane protein, and is the substrate for Aβ production through sequential cleavage. Mutations in the APP gene are associated with the development of genetic forms of AD (Hardy and Allsop, 1991; Hardy and Higgins, 1992). Here, we found APP to be expressed in a neuronal and plaque associated pattern as well as being present intracellularly in primary microglia. Microglial expression of APP has earlier been suggested by a variety of groups (Haass et al., 1991; Paul et al., 1992; Banati et al., 1995), as well as RNAseq data showing expression of APP mRNA in microglial cells *in vivo* (Keren-Shaul et al., 2017). The function of APP expression in microglia is currently unknown but due to its internal location it is less likely to contribute to Aβ metabolism and is more likely to act as an acute phase protein thereby being involved in the inflammatory response. In human tissue, we found APP immunoreactivity to be primarily neuronal and in large aggregates resembling Aβ plaques. Double-IF for CD68 and APP showed CD68^+^ lysosomes and APP to be in close proximity, however, without disclosing the APP producing cell type.

In addition, we found two proteins, APOE and Clu, both being risk factors of sporadic AD (Scheltens et al., 2016), to be increased in Tg mice and CNS myeloid cells from Tg mice as well as being expressed in primary microglia and co-localize to or be co-expressed in CD11b^+^ microglia in Tg mice. As for APP, our data, suggesting APOE and Clu as disease associated proteins in microglia are supported by RNAseq from microglia *in vivo* (Keren-Shaul et al., 2017). APOE and Clu are the two major apolipoproteins in the brain and are known to interact with Aβ and regulate its clearance from the brain through the endothelial low-density lipoprotein receptor related protein 1 and 2 (LRP1/2), respectively (Nelson et al., 2017). In addition, Aβ aggregates are more efficiently taken up and cleared by microglia when Aβ is complexed with lipoproteins including APOE and Clu (Yeh et al., 2016; Ulrich et al., 2018), possibly attributing both proteins a role in the LPS-induced reduction in neocortical Aβ plaque load in the Tg mice. Recently, RNAseq data suggested APOE in combination with TREM2 to induce the microglial phenotypic changes observed in neurodegenerative diseases (Krasemann et al., 2017). In addition, an altered mRNA expression level of APOE, as well as of Ctsz and Hexb in microglia was also suggested to reflect a microglial phenotype associated with neurodegeneration (Keren-Shaul et al., 2017). In post-mortem tissue we found APOE immunoreactivity to resemble what we observed in Tg mice, however, as for APP, using CD68 as a myeloid cell marker, we were unable to determine whether APOE is co-localized to microglia in AD.

Lastly, the Ctsz protein is a CNS-myeloid cell-expressed, and thereby also microglial-expressed, lysosomal protease also known as cathepsin X and has previously been found to be induced by LPS in the microglial cell line BV2 (Pišlar et al., 2017). The study also proposed that Ctsz directly influenced the microglial pro-inflammatory state as its inhibition reduced the LPS-induced production of NO and ROS, and TNF. This, in addition, was compatible with the increased TNF mRNA levels observed in this study in Tg versus Wt mice as well as in response to systemic LPS administration. A substrate of Ctsz is the neurotrophic factor, γ-enolase, whose serum levels may correlate with brain atrophy (Chaves et al., 2010). The neurotrophic activity of γ-enolase depends on an intact C-terminal domain, which is a proteolytic target of Ctsz. Thus increased activity of Ctsz may impair neuronal survival and neuritogenesis (Hafner et al., 2013). In addition, a decreased neuroinflammatory state was observed in a Ctsz knockout mouse with experimental autoimmune encephalomyelitis (Allan et al., 2017), further supporting the involvement of Ctsz in augmenting microglial pro-inflammatory activities, and thereby potentially being a therapeutic target in AD. We observed an altered Ctsz immunoreactivity in AD cortex tissue relative to cognitively healthy controls, with Ctsz immunoreactivity in aggregates of lysosome-sized structures which appeared to be associated with plaques and in microglial-like cells exclusively in AD cases. In addition, we found Ctsz to co-localize with the lysosomal marker CD68 in structures of up to 4–5 μm in diameter, resembling the vacuole-like structures observed along the processes of Iba1^+^ microglial cells in the AD brain, lending additional support to Ctsz being involved in the CNS myeloid cell response to Aβ in AD. Ctsz immunoreactivity in the AD brain has also been reported, however, in less detail, and using a different primary antibody, in a previous report (Wendt et al., 2007). Finally, we observed Ctsz immunoreactivity in perivascular cells in both AD and control groups, which, as the Ctsz expression in microglial-like cells and lysosomes in the AD brain, is a novel observation.

### Strengths and Limitations

In this study, both the Tg and Wt mice were all littermates, of the same gender and housed under identical conditions during their entire lifespan. This gives credibility to our results on LPS effects on cytokines and Aβ pathology in APP_swe_/PS1_ΔE9_ mice, as well as the observations of differentially expressed proteins between genotypes. In addition, the proteins we find differentially expressed in Tg mice and CNS myeloid cells, thus indicating their involvement in AD, also showed differential immunoreactivity pattern in post-mortem cortex tissue from AD cases relative to cognitively healthy controls, supporting their importance in AD pathogenesis.

Further strengthening the results is the fact that the CNS myeloid cells were isolated from 24-month-old Tg and Wt mice. This is a late time-point for any mouse model due to increased mortality at this age as well as cells becoming more fragile during aging (Babcock et al., 2015; Severino et al., 2018). This, however, also set a limitation to the study since the CNS myeloid cells were isolated from naïve mice (not LPS-injected) as well as the primary microglia also being naïve/un-stimulated. Thereby we were unable to describe the direct effect of LPS on CNS myeloid cells in our Tg mouse. In addition, due to the small size of cytokines and their low abundance in complex samples (Nilsson et al., 2010), protein levels of these were not detected by the shot-gun MS strategy used in this study. Thereby cytokine expression affected by LPS was not evaluated on the protein level. Nevertheless, by using this approach we get indications of microglial proteins affected in AD, which is supported by their altered expression pattern in post-mortem cortex tissue from AD cases.

Due to a limitation of available and compatible antibodies as well as the amount of high-quality mouse tissue, we were unable to perform all possible IHC/IF experiments required to support our proteomic results. Thus a Ctsz staining on mouse tissue is not included as well as a Clu staining on human tissue. In addition, to further elucidate on the expression of APP, APOE, Clu, and Ctsz in human microglial cells in AD cases, double-IF of the proteins with a different microglial marker, like Iba1 or ideally a plasma membrane expressed molecule like CD11b, would have been optimal, and should be performed in future studies.

## Conclusion

This study is, to our knowledge, the first comprehensive study of repeated systemic LPS administration in APP_SWE_/PS1_ΔE9_ mice and shows a beneficial effect of LPS administration on Aβ-plaque load, which might be important for the discovery of pathways and new molecular targets with the potential to lower Aβ levels at an early stage of disease. Furthermore this study elucidates on the microglial and perivascular cell lysosomal protein Ctsz, as a novel target in AD pathology. Collectively, these results implicate CNS myeloid cells, especially microglia, in ameliorating amyloid pathology in early-to-mid stage disease in the APP_SWE_/PS1_ΔE9_ mouse and attract attention to the potential disease involvement of Ctsz expressed in CNS myeloid cells in AD.

## Author Contributions

CT: proteomics on hippocampal tissue, IHC and IF staining of tissue and cells, hippocampal plaque-load estimation, *in situ*, qPCR, primary microglia cultures, writing of manuscript. LI: processing of mice, neocortical plaque-load estimation, *in situ*, qPCR, IHC and IF staining of tissue. SK: proteomics on hippocampal tissue. AH: proteomics on CD11b^+^ cells. CvL: primary microglial cultures. AB: processing of mice, isolation of CD11b^+^ cells, guidance/supervision. SD: reviewed the neuropathology of all the cases provided by the Maritime Brain Tissue Bank. ML: proteomics guidance and supervision. BF: initiator of the project, guidance and supervision. All authors read, edited, and approved the manuscript.

## Conflict of Interest Statement

The authors declare that the research was conducted in the absence of any commercial or financial relationships that could be construed as a potential conflict of interest.

## Footnotes

1 http://proteomecentral.proteomexchange.org

## Supplementary Material

FIGURE S1 Proteomics workflows of hippocampal samples **(A)** and CD11b^+^ cells **(B)**.

FIGURE S2 Modified from Ingenuity pathway analysis (IPA) of significantly enriched disease- and biological pathways. **(A)** Significantly differentially expressed proteins between Wt and Tg hippocampal proteomes showed proteins to cluster in disease pathways related to inflammation of the central nervous system and amyloidosis. **(B)** IPA analysis showing significantly enriched biological pathways in Tg versus Wt mice, with and without LPS administration. **(C)** Biological pathways enriched with LPS administration in Tg and Wt mice. White boxes designate non-significant, purple boxes designate significantly enriched pathways with *p* < 0.05.

FIGURE S3 APP, APOE, Clu and Hexb protein expression in Ncx of Wt and Tg mice injected with LPS or PBS (*n* > 2/group) were immunohistochemically stained using primary rabbit antibodies and using an alkaline phosphatase conjugated secondary antibody yielding a bluish-black reaction product. IgG controls showed only vascular signal. Scale bars: 50 μm (low power), 10 μm (high power).

FIGURE S4 **(A)** Aβ (6E10), pTau (AT8) and Iba1 staining in Ncx of AD cases and Iba1 in Ncx of control cases. Scale bars = 100 μm. **(B)** Higher magnification images of Aβ (6e10), pTau (AT8) and Iba1 protein expression in Ncx of AD cases and IBA1 in Ncx of control cases that were immunohistochemically stained. **(C)** APP, APOE, Ctsz, and Hexb protein expression in Ncx of post-mortem AD and control cases. The staining of APP showed neuronal localization (insert) as well as distribution as Aβ-plaque-like structures in AD cases. The APOE staining showed an Aβ-plaque-like distribution in AD cases. The Ctsz staining showed perivascular signal in AD and Control cases (arrows) as well as a cellular signal (arrow heads) in AD cases. The Hexb staining visualized punctate subcellular structures in both AD and control cases. IgG controls showed no staining (Supplementary Figure S5). Scale bars: 50 μm (**A,B**, low power), 10 μm (**B**, inserts), 100 μm (**C**, except insert which is 10 μm).

FIGURE S5 **(A)** Rabbit IgG controls used in the same concentration as for Ctsz. **(B)** Rabbit IgG control used in the same concentration as for Iba1. **(C)** Mouse IgG1 control used in the same concentration as for pTau (AT8) and Aβ (6e10). Scale bar: 100 μm.

FIGURE S6 **(A)** Orthogonal view of Z-stack of mouse tissue shown in Figure 6 stained for APP, APOE, and Clu (green), CD11b (red) and a nuclear counterstain with DAPI (blue). Colocalization was observed (yellow) for APP, APOE, and Clu. The z-stack for Clu had a green signal layer on top, which should be disregarded as the last step of this z-stack included a step outside of the section. **(B)** IgG controls for Figure 6 which has not undergone a deconvolution step. Scale bars: 20 μm, except bottom right corner which is 10 μm.

FIGURE S7 **(A)** Orthogonal view of Z-stacks showed in Figure 7 of PFA-fixed primary microglial cells stained for APP, APOE, Clu, Ctsz, and Hexb (green), CD11b (red) and a nuclear counterstain with DAPI (blue). Intracellular expression is observed for all proteins. **(B)** IgG controls for Figure 7 which has not undergone a deconvolution step. Scale bar: 20 μm.

FIGURE S8 **(A)** Orthogonal view of Z-stack of human tissue shown in Figure 9 stained for APP, APOE, and Ctsz (green), CD68 (red) and a nuclear counterstain with DAPI (blue). Colocalization was observed (yellow) for Ctsz and CD68. **(B)** IgG controls for Figure 9 which has not undergone a deconvolution step. Scale bar: 10 μm.

TABLE S1 Human tissue used for IHC validation of protein targets APP, APOE, Ctsz, and Hexb. Obtained from the Maritime Brain Tissue Bank, Dalhousie University, Halifax, NS, Canada.

TABLE S2 Antibodies and reagents used for immunohistochemistry and immunofluorescence.

TABLE S3 All quantified proteins in the hippocampal proteome and significantly regulated proteins in each condition. (limma test with *q* < 0.01).

TABLE S4 All quantified proteins in the CD11b^+^ cell proteome, significantly regulated proteins between Tg and C57BL/6 CD11b^+^ cells, and proteins overlapping between the CD11b^+^ cell proteome and the hippocampal proteome.
