# Supplementary figures and images for: Diverse Protein Profiles in CNS Myeloid Cells and CNS Tissue From Lipopolysaccharide- and Vehicle-Injected APPSWE/PS1ΔE9 Transgenic Mice Implicate Cathepsin Z in Alzheimer’s Disease
Source: Front Cell Neurosci. 2018 Nov 6;12:397. doi: 10.3389/fncel.2018.00397 (PMC6232379; doi:10.3389/fncel.2018.00397)

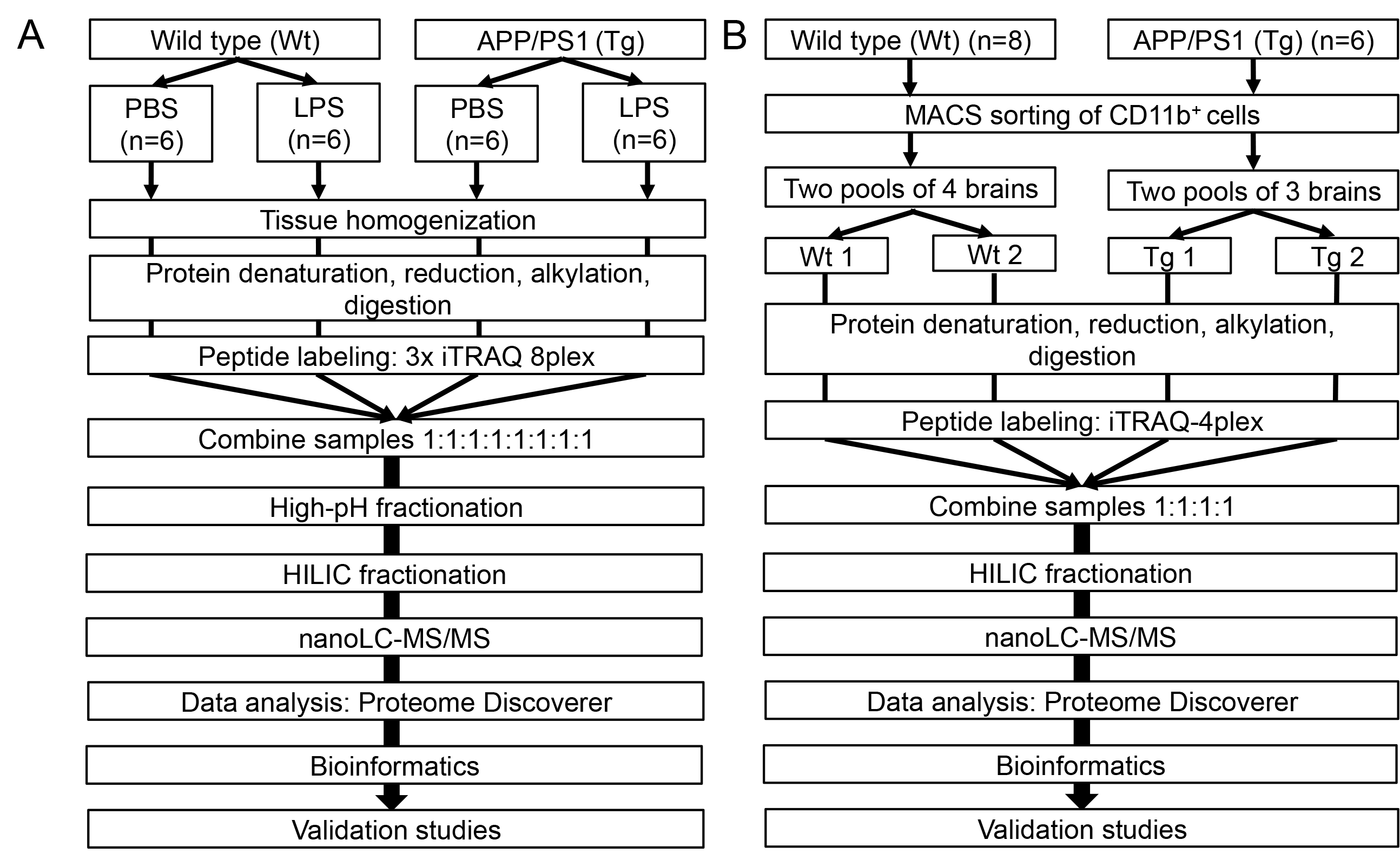

Supplement: FIGURE S1 — Proteomics workflows of hippocampal samples (A) and CD11b+ cells (B). [file Image_1.TIF]

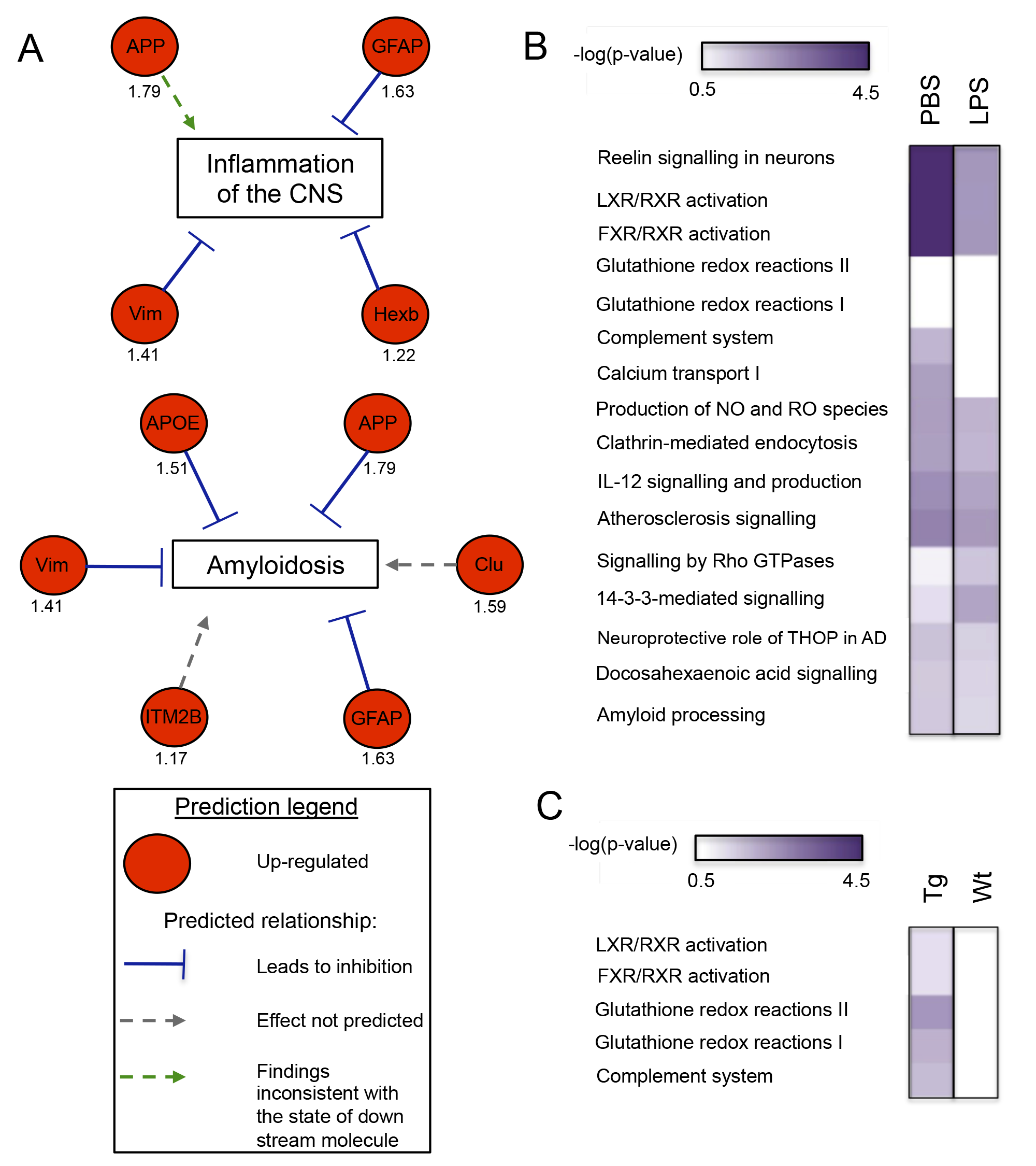

Supplement: FIGURE S2 — Modified from Ingenuity pathway analysis (IPA) of significantly enriched disease- and biological pathways. (A) Significantly differentially expressed proteins between Wt and Tg hippocampal proteomes showed proteins to cluster in disease pathways related to inflammation of the central nervous system and amyloidosis. (B) IPA analysis showing significantly enriched biological pathways in Tg versus Wt mice, with and without LPS administration. (C) Biological pathways enriched with LPS administration in Tg and Wt mice. White boxes designate non-significant, purple boxes designate significantly enriched pathways with p < 0.05. [file Image_2.TIF]

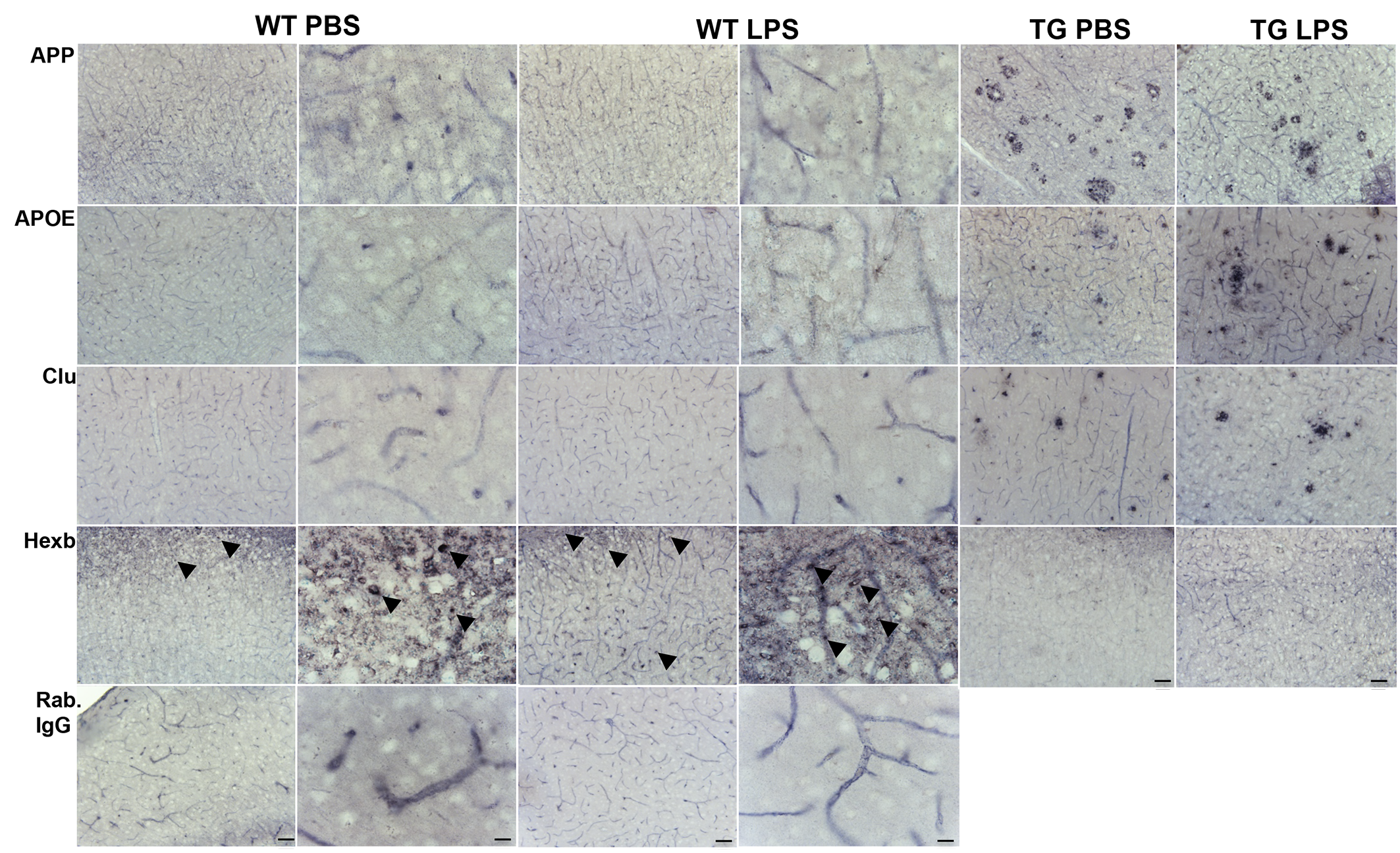

Supplement: FIGURE S3 — APP, APOE, Clu and Hexb protein expression in Ncx of Wt and Tg mice injected with LPS or PBS (n > 2/group) were immunohistochemically stained using primary rabbit antibodies and using an alkaline phosphatase conjugated secondary antibody yielding a bluish-black reaction product. IgG controls showed only vascular signal. Scale bars: 50 μm (low power), 10 μm (high power). [file Image_3.TIF]

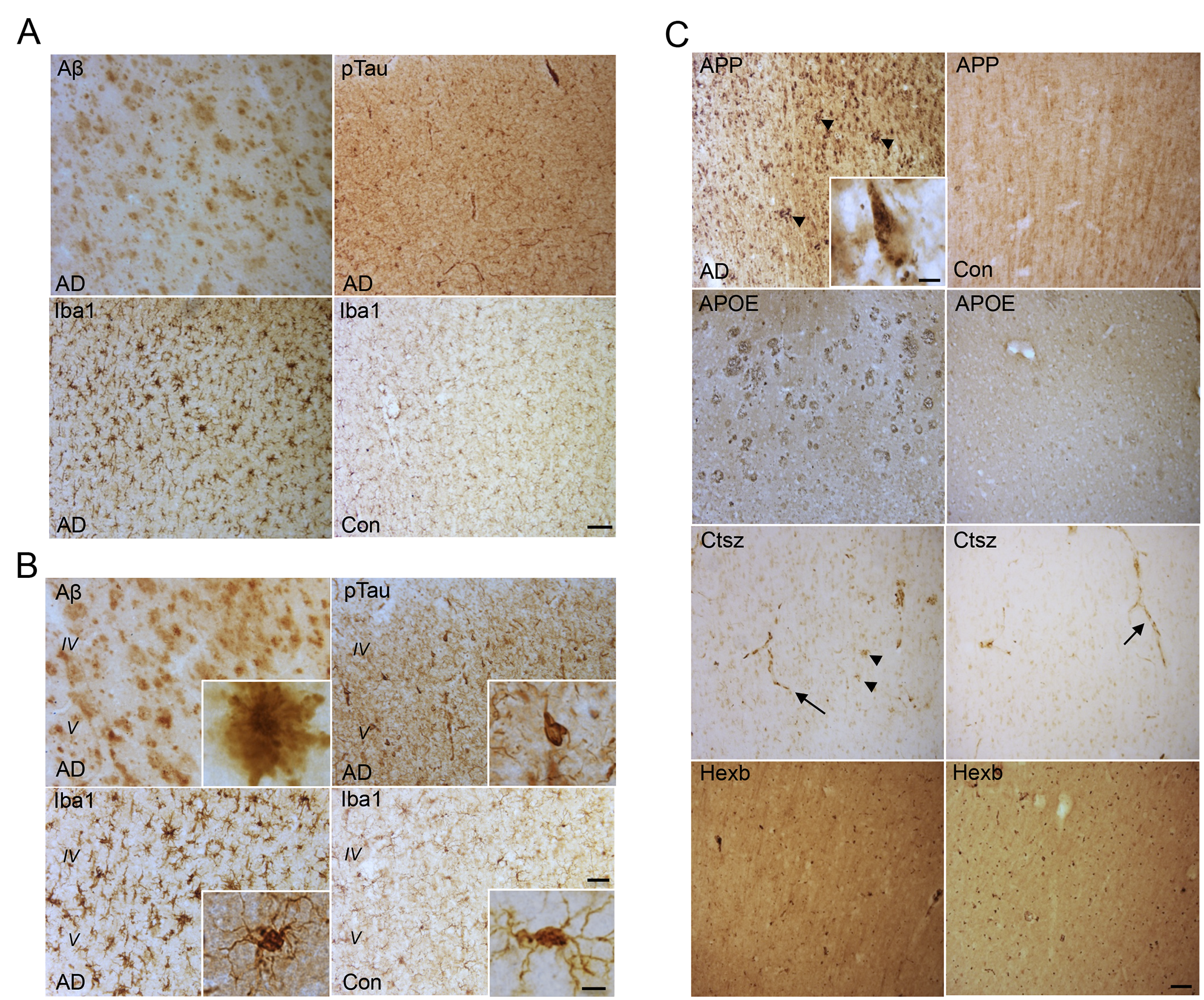

Supplement: FIGURE S4 — (A) Aβ (6E10), pTau (AT8) and Iba1 staining in Ncx of AD cases and Iba1 in Ncx of control cases. Scale bars = 100 μm. (B) Higher magnification images of Aβ (6e10), pTau (AT8) and Iba1 protein expression in Ncx of AD cases and IBA1 in Ncx of control cases that were immunohistochemically stained. (C) APP, APOE, Ctsz, and Hexb protein expression in Ncx of post-mortem AD and control cases. The staining of APP showed neuronal localization (insert) as well as distribution as Aβ-plaque-like structures in AD cases. The APOE staining showed an Aβ-plaque-like distribution in AD cases. The Ctsz staining showed perivascular signal in AD and Control cases (arrows) as well as a cellular signal (arrow heads) in AD cases. The Hexb staining visualized punctate subcellular structures in both AD and control cases. IgG controls showed no staining (Supplementary Figure S5). Scale bars: 50 μm (A,B, low power), 10 μm (B, inserts), 100 μm (C, except insert which is 10 μm). [file Image_4.TIF]

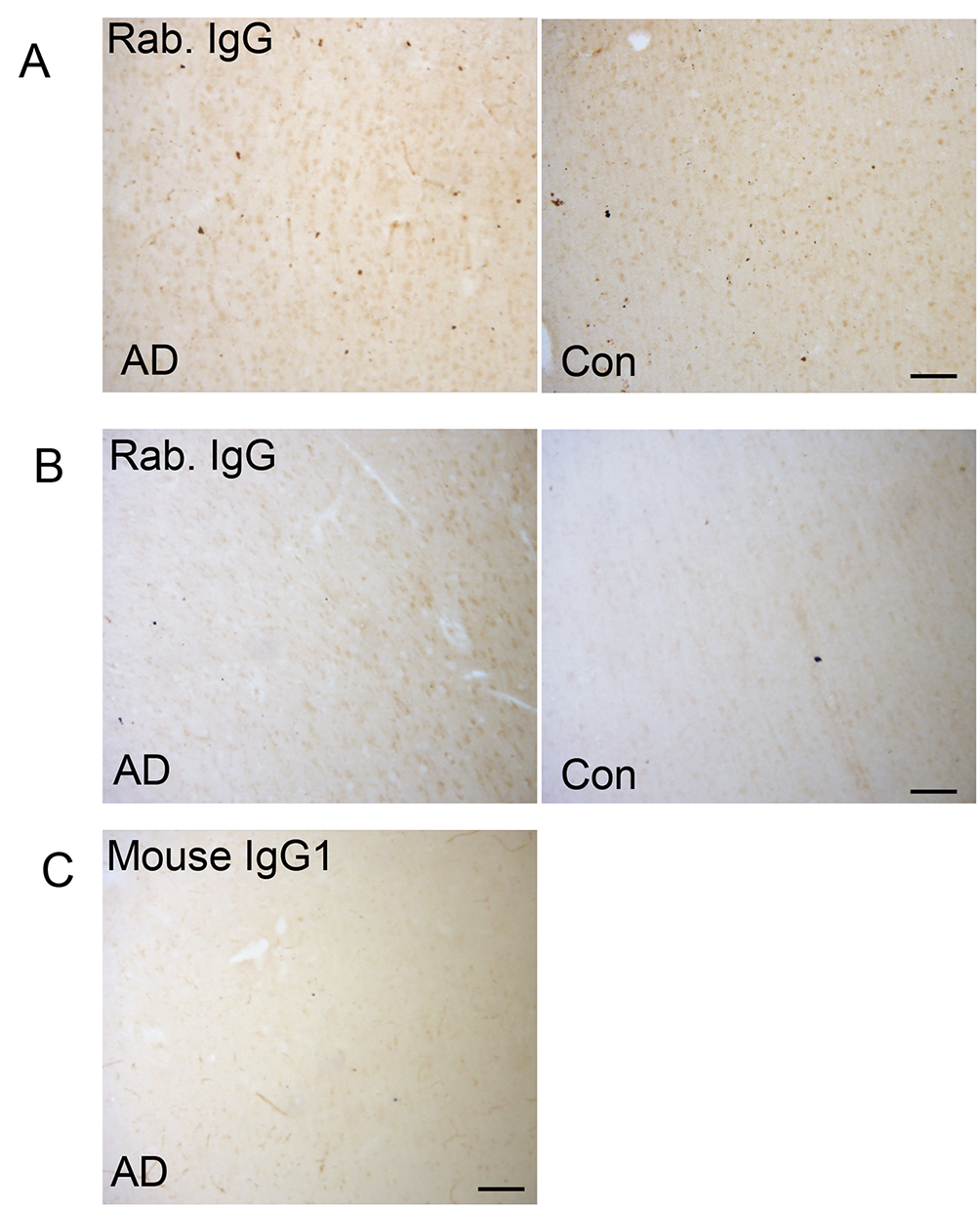

Supplement: FIGURE S5 — (A) Rabbit IgG controls used in the same concentration as for Ctsz. (B) Rabbit IgG control used in the same concentration as for Iba1. (C) Mouse IgG1 control used in the same concentration as for pTau (AT8) and Aβ (6e10). Scale bar: 100 μm. [file Image_5.TIF]

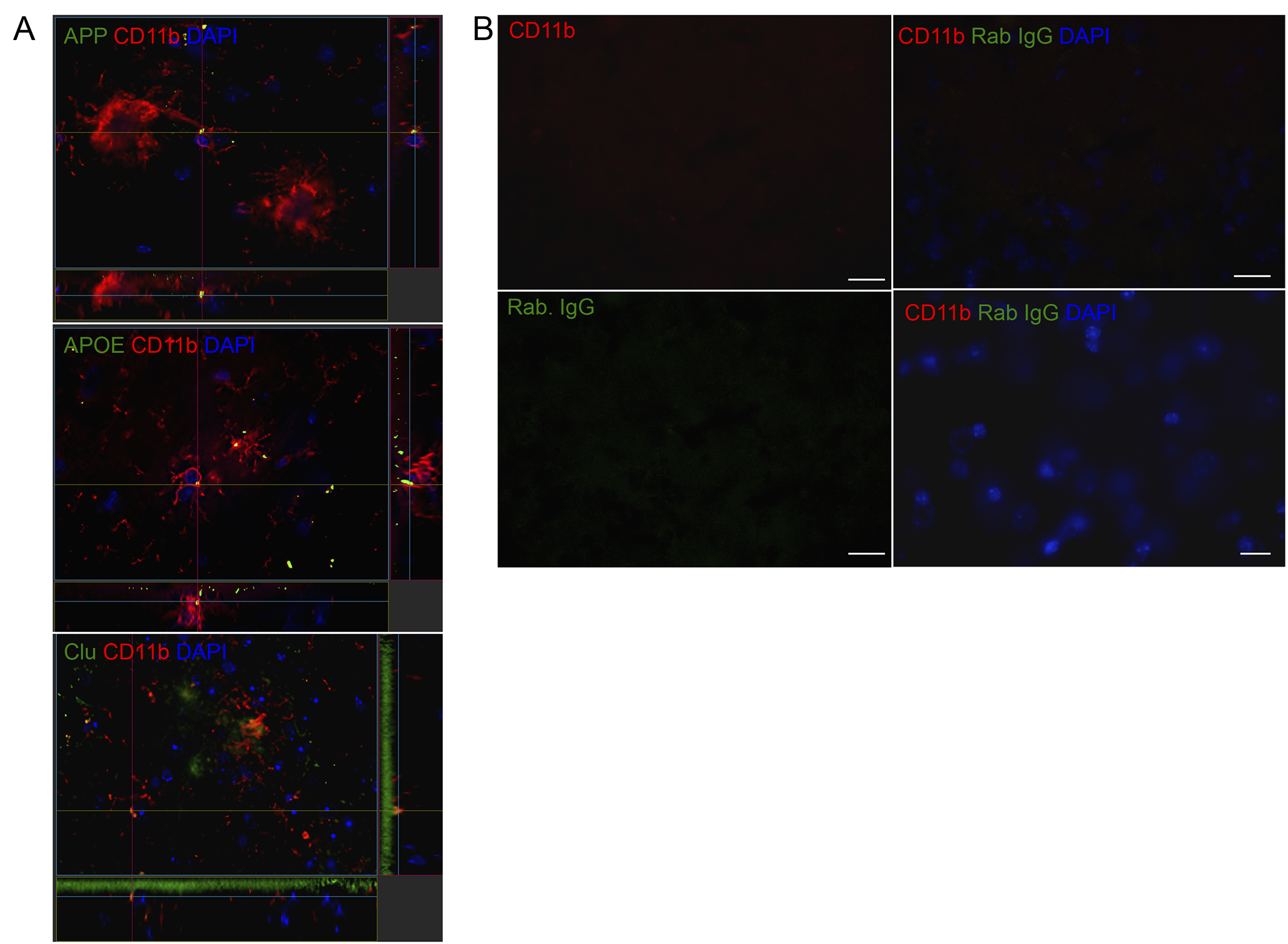

Supplement: FIGURE S6 — (A) Orthogonal view of Z-stack of mouse tissue shown in Figure 6 stained for APP, APOE, and Clu (green), CD11b (red) and a nuclear counterstain with DAPI (blue). Colocalization was observed (yellow) for APP, APOE, and Clu. The z-stack for Clu had a green signal layer on top, which should be disregarded as the last step of this z-stack included a step outside of the section. (B) IgG controls for Figure 6 which has not undergone a deconvolution step. Scale bars: 20 μm, except bottom right corner which is 10 μm. [file Image_6.TIF]

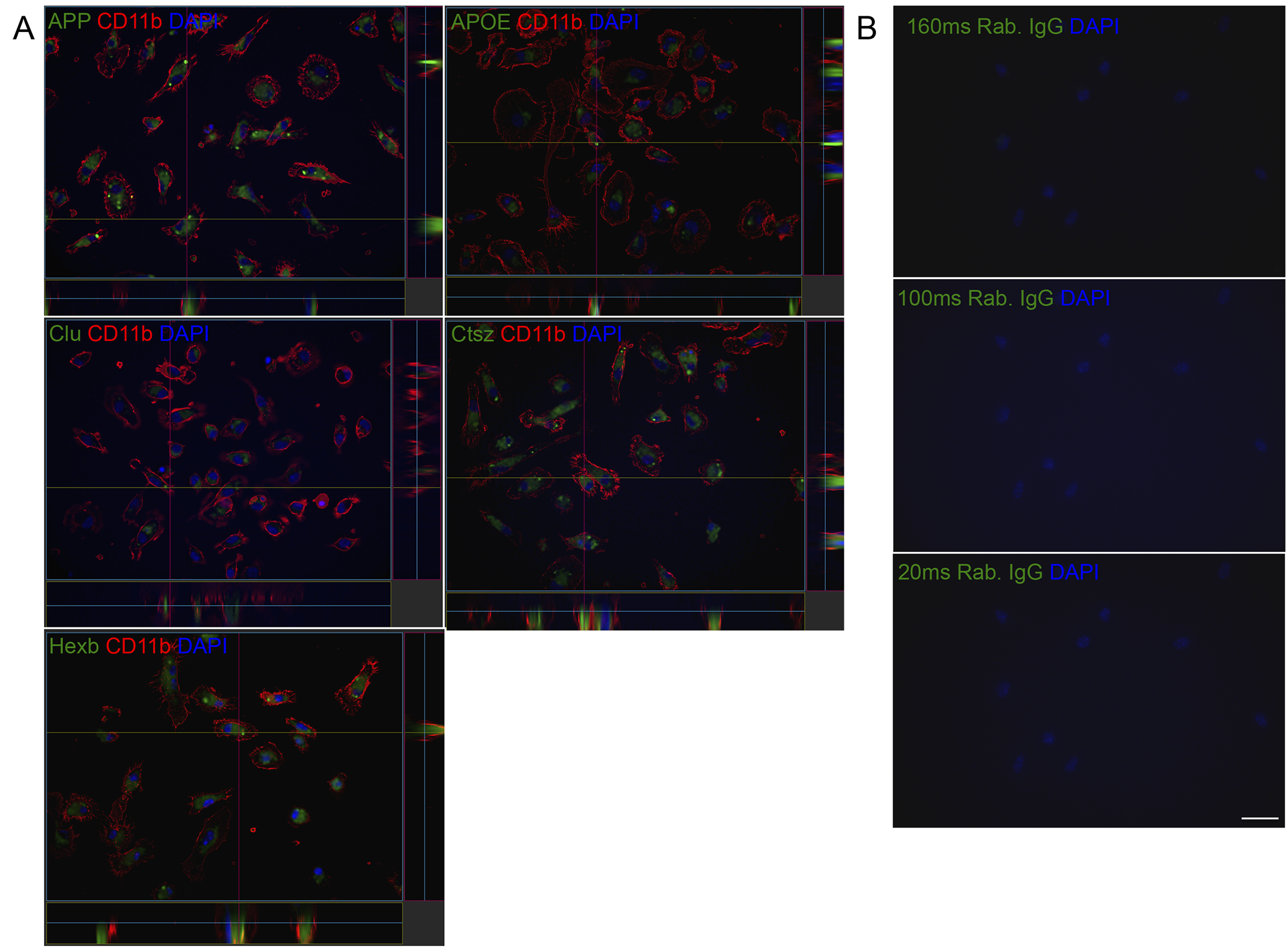

Supplement: FIGURE S7 — (A) Orthogonal view of Z-stacks showed in Figure 7 of PFA-fixed primary microglial cells stained for APP, APOE, Clu, Ctsz, and Hexb (green), CD11b (red) and a nuclear counterstain with DAPI (blue). Intracellular expression is observed for all proteins. (B) IgG controls for Figure 7 which has not undergone a deconvolution step. Scale bar: 20 μm. [file Image_7.TIF]

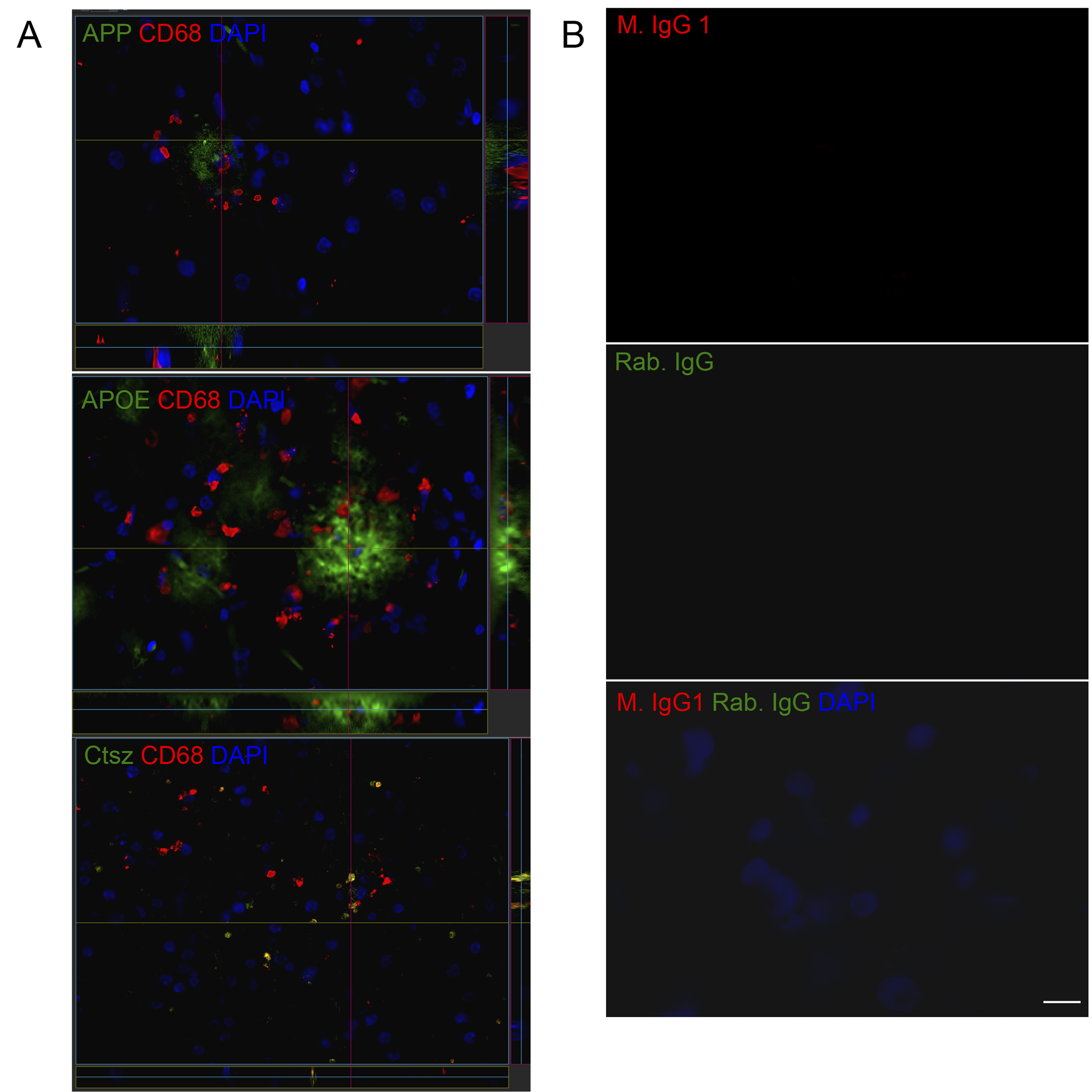

Supplement: FIGURE S8 — (A) Orthogonal view of Z-stack of human tissue shown in Figure 9 stained for APP, APOE, and Ctsz (green), CD68 (red) and a nuclear counterstain with DAPI (blue). Colocalization was observed (yellow) for Ctsz and CD68. (B) IgG controls for Figure 9 which has not undergone a deconvolution step. Scale bar: 10 μm. [file Image_8.TIF]
