# Supplementary material for: Diverse Protein Profiles in CNS Myeloid Cells and CNS Tissue From Lipopolysaccharide- and Vehicle-Injected APPSWE/PS1ΔE9 Transgenic Mice Implicate Cathepsin Z in Alzheimer’s Disease
Source: Front Cell Neurosci. 2018 Nov 6;12:397. doi: 10.3389/fncel.2018.00397 (PMC6232379; doi:10.3389/fncel.2018.00397)
Supplement: TABLE S1 — Human tissue used for IHC validation of protein targets APP, APOE, Ctsz, and Hexb. Obtained from the Maritime Brain Tissue Bank, Dalhousie University, Halifax, NS, Canada. [file Table_1.DOCX]

**Supplementary Table 1:** Human tissue used for immunohistochemical validation of protein targets APP, APOE, Ctsz and Hexb. AD, Alzheimer’s disease; F/M, female/male; NFTs, neurofibrillary tangles; PMI, postmortem interval; Y, years.

| **Disease** | **Sex** (F/M) | **Age** (Y) | **PMI** (Hours) | **Brain Weight** (g) | **Staging** | **Cause of death** |
| --- | --- | --- | --- | --- | --- | --- |
| Definite AD | F | 87 | 7 | 1070 | Definite AD  33 plaques/medium power field  (Scattered NFTs) | Unknown |
| Definite AD | F | 87 | 6.5 | 1280 | Definite AD  39 plaques/medium power field  (Numerous NFTs) | Heart failure |
| Definite AD | F | 81 | 10 | 1250 | Definite AD  35 plaques/medium power field  (Occasional NFTs) | Renal failure |
| Definite AD | M | 86 | 1.75 | 1280 | Definite AD  37 plaques/medium power field  (Frequent NFTs) | Unknown |
| Definite AD | M | 87 | 19 | 1080 | Definite AD  25 plaques/medium power field  (Scattered NFTs) | Unknown |
| Control | F | 90 | 14 | 1030 | Normal  (No history of cognitive impairment)  (Age related few plaques and NFTs) | Pancolitis |
| Control | F | 86 | 17 | 1250 | Normal  (No history of cognitive impairment)  (Age related rare plaques and NFTs) | Heart failure |
| Control | M | 61 | 20 | 1250 | Normal  (No history of cognitive impairment)  (Age related rare plaques and NFTs) | Myocardial infarct |
