# Supplementary material for: Diverse Protein Profiles in CNS Myeloid Cells and CNS Tissue From Lipopolysaccharide- and Vehicle-Injected APPSWE/PS1ΔE9 Transgenic Mice Implicate Cathepsin Z in Alzheimer’s Disease
Source: Front Cell Neurosci. 2018 Nov 6;12:397. doi: 10.3389/fncel.2018.00397 (PMC6232379; doi:10.3389/fncel.2018.00397)
Supplement: TABLE S2 — Antibodies and reagents used for immunohistochemistry and immunofluorescence. [file Table_2.DOCX]

**Supplementary Table 2:** Antibodies and reagents used for immunohistochemistry and immunofluorescence

| **Antibody** | **Company** | **Cat. No.** | **Stock concentration** | **Used concentration** |
| --- | --- | --- | --- | --- |
| Biotinylated mouse anti-human Aβ, clone 6e10 (IgG1 isotype) | Covance | SIG-39340 | 1mg/mL | 2μg/mL |
| Rat anti-mouse/human CD11b, clone 5C6  (IgG2b isotype) | AbD Serotec, | MCA711G | 1mg/mL | 2μg/mL |
| Rabbit anti-mouse/rat/human APOE, clone Y188 | Abcam | Ab183597 | 0.636mg/mL | 2.5μg/mL |
| Rabbit anti-mouse/rat Clu, clone EPR17539-95 | Abcam | Ab184100 | 0.556mg/mL | 2.2μg/mL |
| Rabbit anti-mouse/Rat/human APP, clone Y188 | Abcam | Ab32136 | 0.402mg/mL | 1.6μg/mL |
| Rabbit anti-mouse/human Ctsz, clone EPR14357 | Abcam | Ab180580 | 0.25mg/mL | 2.5μg/mL |
| Rabbit-anti-mouse beta-hexosaminidase (Hexb) | Cloud-clone | PAA637Mu02 | 0.56mg/mL | 2.2μg/mL |
| Biotinylated mouse anti-human pTau, clone AT8 (IgG1 isotype) | Thermo Scientific | MN1020B | 100μg/mL | 0.2μg/mL |
| Rabbit anti-human/mouse/rat Iba1 | WAKO | 019-19741 | 0.5μg/mL | 1ng/mL |
| Mouse anti-human CD68, clone KP1  (IgG1 isotype) | DAKO | M0814 | 425mg/mL | 2μg/mL |
| Rabbit IgG | Dako | XO903 | 20mg/mL | 2.5μg/mL |
| Biotinylated mouse IgG1 | Caltag | MG115 | 4.2mg/mL | 2μg/mL |
| Rat IgG2b | Nordic Biosite | 400602 | 0.5mg/mL | 2μg/mL |
| Mouse IgG1 | DAKO | X0943 | 20g/L | 2μg/mL |
| AlexaFluor-488 goat anti rat IgG | Invitrogen | A-11006 | 2mg/mL | 4μg/mL |
| AlexaFluor 594-labelled goat-anti rat IgG | Invitrogen | A-11007 | 2mg/mL | 4μg/mL |
| SA-TRITC | AbD Serotec | STAR3B | ? | ? |
| AP-conjugated anti-rabbit IgG | Sigma | A3812 | Unknown | 1:200 dilution |
| AlexaFluor 594-labelled goat-anti rat IgG | Invitrogen | A11007 | 2mg/mL | 4μg/mL |
| AlexaFluor 488- labeled Donkey-anti rabbit IgG | Invitrogen | A21206 | 2mg/mL | 4μg/mL |
| DAPI | Invitrogen | D3571 |  | 300nM |
