# Supplementary material for: Diverse Protein Profiles in CNS Myeloid Cells and CNS Tissue From Lipopolysaccharide- and Vehicle-Injected APPSWE/PS1ΔE9 Transgenic Mice Implicate Cathepsin Z in Alzheimer’s Disease
Source: Front Cell Neurosci. 2018 Nov 6;12:397. doi: 10.3389/fncel.2018.00397 (PMC6232379; doi:10.3389/fncel.2018.00397)
Supplement: Supplementary file 13 [file Data_Sheet_1.docx]

**Supplementary methods**

**Mass-spectrometry (MS) based proteomics**

**Isolation of protein:** Snap-frozen hippocampal tissue was homogenized in 6M urea, 2M thiourea, 10mM DTT including protease- and phosphatase inhibitors in a Dounce Homogenizer, and probe-sonicated on ice. 100μg of protein from each sample was used for proteomics and the rest was stored at -20°C for protein validation studies. Protein was isolated from the CD11b^+^ cells by the Qiagen AllPrep kit for simultaneous isolation of protein, RNA and DNA, and concentrated on a 10kDa spin filter with additional washing steps using 50nM TEAB to remove detergent from the elution buffer. Protein samples were denatured and reduced in 6M urea, 2M thiourea, 10mM DTT at room temperature (RT) followed by probe-sonication of the hippocampal proteins. Denatured proteins were hereafter alkylated in 20mM iodoacetamide (IAA) for 20 min in the dark.

**Enzymatic digestion:** Subsequently, a total of 2μl of endoproteinase Lys-C (6μg/μl, Walko) was added to the hippocampal protein samples and incubated 2 hours at RT. Samples were diluted 10 times with 20mM TEAB, pH 7.5, and digested with trypsin (1:50 (w/w) trypsin:protein) ON at RT. The enzymatic digestion was stopped with 5% formic acid (FA) and peptide samples were cleared by centrifugation (14000 x g, 15 minutes). CD11b^+^ cell proteins were digested with 1μl of endoproteinase Lys-C (6μg/μl, Walko) for 1 hour at RT. Samples were diluted 4 times with 50nM TEAB, pH 7.5, and digested with 5μg trypsin ON at RT. CD11b^+^ cell peptides were subjected to delipidation where samples were dried down and re-suspended in 90% acetonitrile (ACN), 0.1% trifluoroacetic acid (TFA) and run over an in-house prepared column with TSKgel amide-80 (2nm, 3μm particle size¸ Tosoh Bioscience) equilibrated with 90% ACN, 0.1% TFA. Peptides were eluted slowly with 0.1% TFA and the eluate was dried down prior to iTRAQ-4plex labeling. Protein and peptide quantification was performed by fluorometric quantification (Qubit^TM^ – Life technologies).

**iTRAQ labeling:** A total of 100μg tryptic peptides per hippocampal sample were dried and desalted with R2/R3 columns (as described under sample desalting with R2/R3 microcolumn) before iTRAQ-8plex labeling (AB Sciex). A total of 24 mice (n=6/group) were used for the proteomics analysis of the hippocampus, resulting in six biological replicates, which were labeled using three iTRAQ-8plex kits according to the manufacturer’s instruction. The labeled peptides from each sample were mixed 1:1:1:1:1:1:1:1 (Figure 1A), dried by vacuum-centrifugation and stored for further analysis at -80°C. The peptide samples from the CD11b+ cells were labeled with iTRAQ-4plex and mixed 1:1:1:1 (Figure 1B).

**Sample desalting with R2/R3 microcolumn:** Before high-pH Hydrophilic interaction chromatography (HILIC) samples were desalted using P200-tip-based columns packed with equal ratios of Poros R2 (Oligo R2 Reversed Phase Reson 1-1112-46, Applied Biosystems) and Poros R3 (Oligo R3 Reversed Phase Reson 1-1339-03, Applied Biosystems) reversed-phase material. The tip was blocked with C_8_ material (Moden 2314, 3m Empore TM C8). The column was prepared by centrifugation (1000xg) of the R3 reversed-phase resin (100% ACN). The column was equilibrated with 0.1% TFA and the acidified samples were loaded and washed three times with 0.1% TFA. Peptides were eluted with 60% ACN, 0.1% TFA and dried by vacuum centrifugation.

**High-pH fractionation:** Hippocampal peptide samples were fractionated using high-pH fractionation. This was done using R2/R3 microcolumns, as described above. Columns were equilibrated in NH_3_, pH 10, and samples were loaded, washed with 0.1M NH_3_, and eluted with increasing concentrations of ACN/0.1% NH_3_ in steps of 5, 10, 13, 15, 17, 20, 25, 30, 40 and 60% ACN and dried by vacuum centrifugation.

**HILIC fractionation:** High-pH fractions and CD11b^+^ samples were fractionated using HILIC as described in (McNulty and Annan, 2008; Melo-Braga et al., 2015). Peptides were dissolved in 90% ACN, 0.1%TFA (solvent B) and loaded onto a 450μm OD x 320μm ID x 17cm micro-capillary column packed with TSK Amide-80 (30μm; Tosoh Bioscience) using an Agilent 1200 Series HPLC. Peptides were separated using a gradient from 100-60% solvent B (A= 0.1% TFA) in 30 min at a flow-rate of 6μl/min. Fractions were collected every minute based on the UV chromatogram. Subsequently, the peptide fractions were dried by vacuum centrifugation.

**Reversed phased nanoLC-ESI-MS/MS of hippocampal samples:** Fractionated peptides were re-suspended in 0.1% TFA and automatically injected and loaded on a ReproSil-Pur C18 AQ (Dr. Maisch, Ammerbuch, Entringen, Germany) trap column (2cm x 100μm inner diameter; 5μm). The peptides were separated at 250nl/min on an analytical ReproSil-Pur C18 AQ column (17cmx75μm; 3μm) by reversed-phase chromatography, which was operated on an EASY-nanoLC system (Thermo Fischer Scientific, Odense, Denmark). The mobile phase was 95% ACN/0.1% FA (B) and water/0.1% FA (A). The gradient was from 1% to 30% solvent B in 80 min, 30-50% in 10 min, 50-100% B in 5 min and 8 min at 100% B. The nano-LC was online connected to an Orbitrap Fusion tribrid mass spectrometer (Thermo Fischer Scientific) operating in positive ion mode and using data-dependent acquisition. The orbitrap acquired the full MS scan with an automatic gain control (AGC) target value of 3x10^6^ ions and a maximum fill time of 100ms. Each MS scan was acquired at high-resolution (120,000 full-width half maximum (FWMH) at m/z 200 in the orbitrap with a mass range of 400-1400 Da. The 12 most abundant peptide ions were selected from the MS for higher energy collision-induced dissociation (HCD) fragmentation collision energy: 34V (>doubly charged). Fragmentation was performed at high resolution (60,000 FWHM) for a target of 1x10^5^ and a maximum injection time of 60ms using an isolation window of 1.2m/z and a dynamic exclusion of 20s.

**Reversed-phased nanoLC-ESI-MS/MS of CD11b^+^ samples:** Samples were analyzed as described in the previous section with few modifications. In brief, peptides were re-suspended in 0.1% TFA, and automatically loaded onto a ReproSil-Pur C18 AQ in-house packed trap column (2cm x 100μm inner diameter; 3μm). Peptides were separated on an analytical ReproSil-Pur C18 AQ in-house packed column (17cmx50μm; 3μm) by reversed-phase chromatography, which was operated on an EASY-nanoLC system (Thermo Fischer Scientific, Odense, Denmark). The mobile phase was 90% ACN/0.1% FA (B) and water/0.1% FA (A). The gradient was from 1% to 34% solvent B in 60 min. The nano-LC was online connected to an Orbitrap Fusion tribrid mass spectrometer (Thermo Fischer Scientific) operating in positive ion mode and using data-dependent acquisition. The instrument was run in High-High mode with a resolution of 60,000Da and the top N peaks were chosen for HCD fragmentation every 3 second, with normalized collision energy of 42. The orbitrap acquired the full MS scan with an AGC target value of 5x10^5^ ions and an MS/MS AGC target value of 2x10^4^, the maximum fill time was 100ms and ions were selected with a threshold intensity of 10000.

**Data analysis of hippocampal raw data:** Raw LC-MS/MS data was searched against the Swissprot (16,968 reviewed entries) and Uniprot mouse reference database (53,106 entries) via Mascot (v2.3.02, Matrix Science) and Sequest HT search engines, respectively, using Proteome Discoverer (V1.4.1.14, Thermo Fischer Scientific). A precursor mass tolerance of 10ppm and a product ion mass tolerance of 0.02Da were applied allowing not more than one missed cleavage for trypsin. Fixed modifications included carbamidomethylation of Cys and iTRAQ8-plex labeling for Lys and N-termini. Quantification was performed on the centroid peak intensity with the “reporter ions quantifier” node. The Mascot Percolater algorithm (q-value filter: 0.01) was used to ensure a high-confident identification of peptides. In addition, Mascot and Sequest HT peptide rank 1, Mascot score >22 and Sequest HT ΔCn of 0.1 was applied. Moreover, a cut-off value of Xcorr score for charge states of +1, +2, +3 and +4 higher than 1.5, 2, 2.25 and 2.5, respectively, were considered for further analysis. Subsequently peptides were filtered against a Decoy database resulting in a false discovery rate (FDR) of 0.01 (FDR<0.01). Proteins were identified with at least 2 unique peptides. Six biological replicates were considered for the statistical analysis. The statistical analysis was performed on log2 transformed quantification values by a moderated t-test (limma) using rank products, with a q-value threshold of 0.01, corrected for statistical error by means of multiple testing (Schwämmle et al., 2013). The mass spectrometry proteomics data have been deposited to the ProteomeXchange Consortium ([**http://proteomecentral.proteomexchange.org**](http://proteomecentral.proteomexchange.org/)) via the PRIDE partner repository (Vizcaíno et al., 2013) with the dataset identifier < PXD005785 >. (Reviewer account details: username: reviewer45188@ebi.ac.uk; password: rEG8eJzw).

**Data analysis of CD11b^+^ cell raw data:** Raw LC-MS/MS data was searched and filtered as described above. Significantly regulated proteins were determined based on the expression ratio being outside two standard deviations of the biological replicates thereby cut off values were set to 1.3 for up-regulated and 0.75 for down-regulated. The threshold criteria for the ratios were calculated on the average experimental technical variance of the multiple analysis of brain technical replications in mass spectrometry labeling analysis and has successfully been applied in proteomics studies using various mammalian brain regions (Kempf et al., 2014, 2016).
